# Relationships between anxiety, depression and wound healing outcomes in adults: A systematic review and meta-analysis

**DOI:** 10.1371/journal.pone.0309683

**Published:** 2025-05-20

**Authors:** Fiona O’Donovan, Lora Capobianco, Joseph Taylor-Bennett, Adrian Wells

**Affiliations:** 1 Division of Psychological Sciences, School of Health Sciences, Faculty of Biology, Medicine and Health, The University of Manchester, Manchester, United Kingdom; 2 Psychosocial Service, Manchester University NHS Foundation Trust, Manchester, United Kingdom; 3 Department of Research and Innovation, Greater Manchester Mental Health NHS Foundation Trust, Manchester, United Kingdom; 4 Manchester Specialist Psychotherapy Service, Greater Manchester Mental Health NHS Foundation Trust, Manchester, United Kingdom; 5 Greater Manchester Mental Health NHS Foundation Trust, Manchester Academic Health Science Centre, Manchester, United Kingdom; IRCCS Istituto Romagnolo per lo Studio dei Tumori Dino Amadori / Skin Cancer Unit, Romagna Institute for the Study of Cancer IRST IRCCS Dino Amadori, ITALY

## Abstract

**Objectives:**

To examine whether there is a relationship between anxiety and/or depression and wound healing.

**Design:**

Systematic review and meta-analysis.

**Data Sources:**

Searches were conducted on PsycINFO, MEDLINE, EMBASE, CINAHL and Web of Science on the 06-March-2023.

**Methods:**

Eligible studies explored the effects of anxiety and/or depression on wound healing in adults. Healing outcomes included time to heal and complication rates. Anxiety and depression outcomes were considered separately.

**Results:**

Fifty-five studies were included in the narrative synthesis (26,612,809 participants), and 26 studies in the meta-analysis. Studies utilised a range of observational and experimental designs. Wounds included in the review were: surgical, ulcer, burn and experimental wounds. The narrative synthesis gave mixed results, with some studies noting positive associations between increased anxiety or depression and wound healing, while others did not find an association. Results from the meta-analysis found no significant effect of anxiety on wound healing outcomes. However, depression was associated with significantly higher odds of delayed wound healing, OR = 2.10, [1.02, 4.33]; higher risk of wound complications, RR = 1.30, [1.11, 1.53] and increased risk of wound infection RR = 1.25, [1.09, 1.44].

**Conclusion:**

These findings suggest depression negatively impacts wound healing. There is less evidence for an association with anxiety, but this may be due to less research in this area. Future studies should explore the mechanism of associations between depression and wound healing to inform clinical interventions.

## Introduction

Wound healing is crucial in recovery from injury and surgical wounds. Wound management presents a significant clinical, economic, and social burden and in the UK costs the NHS an estimated £8.3 billion per year [1]. Delayed healing is associated with increased pain, psychological distress, reduced mobility and social isolation [2]. Wound healing is a complex, dynamic, multi-stage process influenced by multiple factors. While the physical health variables that influence healing are relatively well understood (e.g., age, wound type, chronic health conditions; [3]) there is a growing acknowledgement of the impact of psychological factors on healing [4].

Studies suggest common mental health difficulties, such as anxiety and depression may impact the wound healing process in a number of ways. Anxiety and depression can trigger the physiological stress response. This response activates the sympathetic-adrenal-medullary (SAM) and the hypothalamic-pituitary-adrenal (HPA) axes. The SAM axis triggers the release of catecholamines such as noradrenaline and norepinephrine, while the HPA axis secretes glucocorticoids such as cortisol [5]. There is considerable evidence from experimental studies (human and animal) that catecholamine and glucocorticoid production slows wound healing [4]. Moreover, neuropeptides oxytocin and vasopressin have been considered regulators of anxiety and depression symptoms [6], and have also been implicated in wound healing [7], therefore such neuropeptides might be a pathway through which psychological factors impact healing. However, it is also likely that psychological factors may impact through health behaviours. Individuals experiencing adverse emotional states such as anxiety or depression may be more likely to consume alcohol and tobacco, make poor dietary choices, experience poor sleep, and engage in low levels of exercise, all of which have been associated with slower wound healing [8–12].

An issue with existing research is that it often lacks specificity when exploring relationships between psychological factors and wound healing. For example, a previous systematic review looked at the effect of psychological stress defined as “any form of negative psychological state, condition, or experience” ([13] p. 254). While this definition was chosen to reflect the variability in the published literature, such a broad definition may mask differential effects of different psychological states and conflates the concept of stress with emotion.

Given such important limitations, we aimed to examine the effects specifically of negative emotions of anxiety and depression on wound healing, since these are common responses in physical health settings that might be managed through psychological treatment methods. Consequently, the aim of this review is to address the question: do anxiety and/or depression have an effect on wound healing?

## Methods

### Protocol details and reporting guidelines

The protocol for this review was registered with the International Prospective Register of Systematic Reviews (PROSPERO; ID CRD42021269269). The paper is reported with reference to the Preferred Reporting Items for Systematic Review and Meta-Analysis Protocols (PRISMA) statement [14]. Please see S1 Table for the PRISMA checklist. No ethical approval was requested as the review utilised data from previously published studies in which informed consent was obtained by the primary investigators.

### Search strategy

A systematic review was conducted in March 2023 of the following electronic databases: PsycINFO, MEDLINE, EMBASE, CINAHL and Web of Science Search terms were agreed with the authors (FOD, LC, AW). The search strategy included terms relating to anxiety, depression and wound healing. The full search strategy is displayed in S2 Table. Further studies were identified from reference lists of related studies.

### Eligibility criteria

Studies were eligible if they were published in a peer-reviewed journal, evaluated wound healing in adult humans and included a validated measure of anxiety and/or depression. Only English language quantitative studies were eligible for inclusion. Studies reported as abstracts in conferences, theses and book chapters were excluded. Similarly, reviews and meta-analyses were excluded.

For this review, a “wound” was defined as a ‘disruption of normal tissue structure and function’ [13]. This encompassed a range of wound types, including clinical wounds (e.g., burns injuries, ulcers, surgical wounds) and experimentally induced wounds (e.g., punch biopsy wound and suction blister). As the outcome of interest was rate of wound healing studies had to include an outcome on wound healing. This included time to heal or whether a wound is classified as healed or not by a time point. It also incorporated indirect measures of wound healing, such as rates of wound complications, wound infections, or wound dehiscence (when a surgical incision wound reopens post operatively).

Psychological factors of interest were anxiety and depression using self-report measures or diagnostic criteria. Anxiety included: anxiety symptoms, anxiety diagnoses, trait anxiety, state anxiety, worry and neuroticism. Depression included depressive symptoms, depression diagnoses, low mood and negative affect. Studies that only included measures of related but distinct concepts (e.g., general distress, stress, or quality of life) were excluded.

### Selection process

Searches were saved to EndNote 20 to create a master file of all references. Duplicates were removed using the process described by Bramer and colleagues [15]. All titles and abstracts were screened by one reviewer (FOD) to determine potential eligibility. Full texts were then screened for eligibility by one reviewer (FOD). A second reviewer (JTB) independently screened 20% of the full texts to check for consistency. There was a moderate agreement between the two reviewers (k = 0.46).Disagreements were resolved by discussion.

### Data collection process and data items

Data extraction was conducted by the first author. Study characteristics were extracted which included: study citation, year of publication, study location, setting, design, participant details (i.e., number of participants, gender and age), measure of anxiety and/or depression, details of wound (e.g., punch biopsy, surgical wounds), wound healing outcome, and key results. Any unclear or missing data was documented in the extraction form.

### Quality appraisal

Risk of bias of included studies was assessed by the first author using the Effective Public Health Practice Project (EPHPP) Quality Assessment Tool for Quantitative Studies [16]. This tool was selected because it has good reliability and validity and can be applied to different study designs [17]. Risk of bias was assessed at the study level. No studies were excluded based on their quality appraisal rating. No weighting was provided to studies based on quality ratings.

### Data synthesis

All included studies were incorporated into a narrative summary and tabulation of findings. Tables were grouped by wound types. Narrative syntheses were grouped a) by psychological variable (anxiety or depression) and b) by wound outcome measure.

Where possible studies were included in a meta-analysis. Meta analyses were conducted on *R* [18] using the *meta* package [19], as guided by Harrer and colleagues [20]. All analytic code and data is available to view/download from Open Science Framework (https://osf.io/m9nre/). Studies were pooled and analysed using a random-effects (RE) model to obtain the summary effect estimates and forest plots were created. Heterogeneity between studies was explored through visual inspection of the forest plots and using the I^2^ statistic. When clarification on heterogeneous data was required, authors were contacted.

Meta-analyses were conducted separately for different wound healing outcomes (e.g., healing time combined separately from infection rate). Where possible data were converted to common metrics to be combined, except in cases where there was insufficient data or measures were conceptually distinct (i.e., studies using Hazard Ratios (HRs) or Odds Ratios (ORs); see Harrer et al [20]). In order to convert HRs and ORs to similar log scales and calculate standard error, the Revman Calculator function was used [21]. In addition, some studies calculated the odds of wound healing [22], whereas others calculated the odds of *not* healing [23]. In these scenarios, some of the ratios were inverted to facilitate meta-analytic synthesis.

A number of included studies analysed data from the same large-scale databases, albeit using different time windows. In this scenario, both studies were described in the narrative synthesis. However, in the meta-analyses if more than one study reported data from the same database, examining the same wound type, only the study with the largest sample size was included in the meta-analytic synthesis, to avoid double counting of participants. See comment by Tarp et al., for a brief discussion on analytical issues presented when combining multiple analyses of the same database [24]

## Results

### Study selection

**Fig 1:**
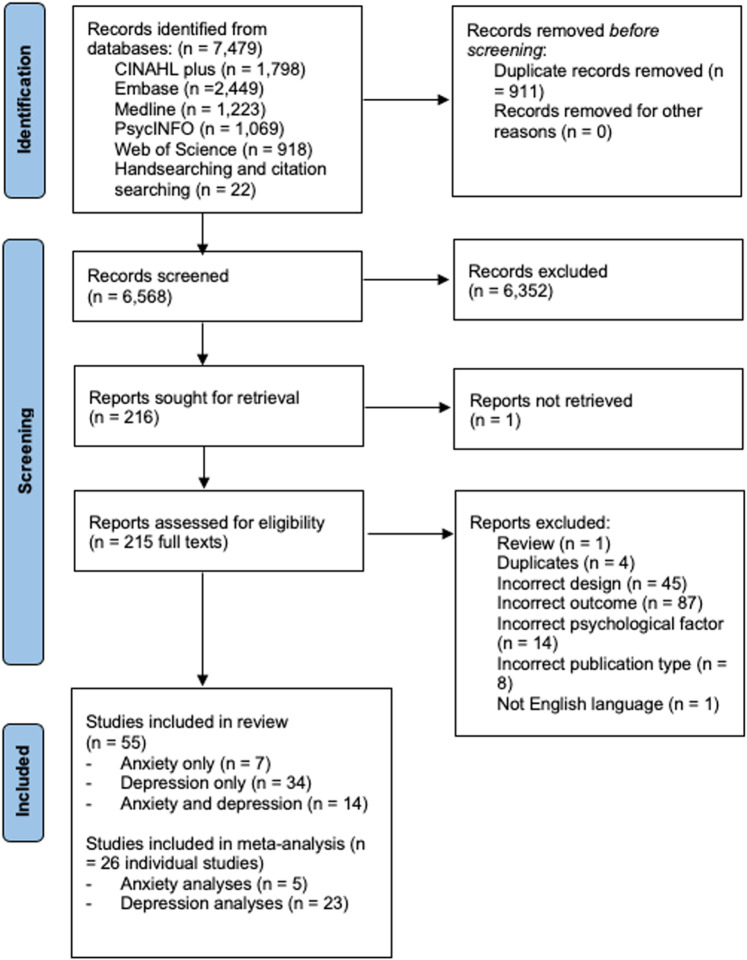
PRISMA flow diagram of study reports eligible for inclusion.

A flow diagram outlining the study selection process is outlined in Fig 1. From the database searches and journal hand searching, 7,479 records were identified. Duplicates were removed, leaving 6,568 records remaining. Following title and abstract screening, 6,352 records were excluded, and 216 records remained for full text screening. All except one full text were obtained. Fifty-five studies were included in the review, seven of which explored the impact of anxiety only, 34 examined the effects of depression only, and 14 looked at the impact of both anxiety and depression. Of the 55 studies included in the review, 26 were included in the subsequent meta-analyses. Wherein published papers included reports of multiple samples, the sample relevant to the current research question were described [25]. Likewise, features of the design that are relevant to the current research question were described. For example, Monami [26] and colleagues describe a study that has an overall follow up time of 12 months, however, only a 6-month follow-up period was used for their analysis on wound healing, therefore the study is described using a 6-month follow up period throughout the review. Some studies employed interventions in certain groups of participants but analysed the association between psychological factors and wound healing for the whole study population. These studies were considered eligible for inclusion [27–29]. One study that was initially selected reported unusual results of no wound complications in large samples of anxiety (n = 139,267) or depression (n = 342,769) patients following surgery [30]. The authors were contacted, and they explained that their study only assessed complications at time of surgery and not follow up and they explained that their study did not assess wound healing [30]. Therefore, the study in question was not included in the present review.

### Description of included studies

The studies included various types of wounds. Thirty studies examined healing of surgical wounds including: ankle surgery [31,32], knee or hip arthroplasty [33–39], total shoulder arthroplasty [40,41], spinal surgery [42–44], dental procedures [45–47], cardiac surgeries including Coronary Artery Bypass Graft (CABG, [48–52]) or other mixed surgeries [53–60]. Fourteen studies examined healing of various types of ulcers, namely: leg ulcers [61–67], foot ulcers [22,26], duodenal ulcer [25,68], or mixed ulcers [23,69,70]. Two studies looked at healing in burn wounds [71,72]. Nine studies created wounds experimentally to track healing, these wounds included suction blister wounds on participants’ forearms [27,73,74], punch biopsy wounds on participants’ arms [28,75,76], a circular wound created on participants’ oral hard palate [77] and a tape stripping paradigm wherein skin barrier function was disrupted using tape stripping procedure on participants” arms. Rate of recovery was then measured using Transepidermal Water Loss (TEWL; [29,78]). TEWL provides a measure of the skin’s ability to prevent water loss. TEWL decreases as the skin barrier is restored, thereby giving an objective measure of skin barrier recovery rate following skin disruption/wounding.

Twenty-seven of the studies utilised prospective designs and monitored healing for a period of time ranging from 2-hour follow up of experimentally induced skin injury [29,78] to tracking ulcer healing for up to 1.5 years [70,79]. Nineteen of the studies employed retrospective cohort designs. Two studies used retrospective chart reviews and a single study utilised a retrospective case control design [71]. Three of the included studies were cross sectional and three were randomised controlled trial (RCT) designs.

Study sample sizes ranged from 17 to 8,710,630 participants. Across all studies, there was a total sample size of 26,612,809. However, it is not possible to verify that these are all unique individuals. Various studies used large scale databases such as the Truven Marketscan database (albeit using different time windows) therefore it is possible that some participants could be double counted. Not all studies reported the ratio of participants’ gender, but of those that did, across all studies 58.18% of participants were female. Similarly, not all studies reported mean age of participants, but in studies that did report this, the mean age range per study was 20.1 [78] to 79.7 [44].

Further detail on the characteristics and results of included studies are displayed in Table 1 for surgical wounds, Table 2 for ulcer and burn wounds, and Table 3 for experimental wounds.

**Table 1:** Characteristics and results of studies of surgical wound studies.

Authors, year Country	Surgery type; Study design characteristics;	Participants: n; age ± SD; % female	Anxiety or depression measure	Wound healing outcome(s)	Key findings	Quality rating
**Ankle surgery**						
Broggi et al., 2022 ^†^ USA	Ankle fracture surgery; Retrospective cohort study using the Truven MarketScan Database between 2009 and 2018	Control group: 93,916; 49.3 ± 16.8; 59.7% Depression group: 13,981; 51.7 ± 15.1; 77.7%	Depression diagnosis (preoperative)	Wound complications	Depressed patients had higher incidence and odds of wound complications compared to control patients, 3.1% vs 2.3%, OR = 1.13, 95% CI [1.00–1.28], p < .001	Strong
Wilson et al., 2022 ^†^ USA	TAA; Retrospective cohort study using the Nationwide Readmission Database between 2011 and 2016	Control group: 7,129; most common age range: 65–74; 46.2% Depression group: 204; most common age range: 65–74; 69.2%	Depression diagnosis (preoperative)	-Any wound complications -PJI -SSI -Wound dehiscence	Depressed patients had increased odds of -any wound complications, OR = 1.59 [1.11, 2.29], p = 0.12; -PJI OR = 1.82 [1.06, 3.15], p = 0.31; -SSI OR = 1.62 [1.02, 2.58], p = 0.41; but not wound dehiscence OR = 1.58 [0.93, 2.58], p = 0.09	Strong
**Knee/hip Arthroplasty**						
Bozic et al., 2012 USA	Primary TKA; Retrospective cohort study using the Medicare 5% sample between 1998 and 2007	83,011, age 65 + , NR	Preoperative depression was identified from Medicare claims forms submitted in the 12-months before surgery	PJI 90 days post operation	Depression associated with increased risk of PJI, adjusted HR = 1.28, [1.08, 1.51], Wald χ2 = 8.53, p = .004	Strong
DeGouveia et al., 2022 USA	Primary THA; Retrospective cohort study using the Humana claims database (PearlDriver) between 2005 and 2014	Matched control group: 115,015; most common age range: 65–69; 61.66% Depression group: 23,061; most common age range: 65–69; 61.63%	Depressive disorder diagnosis (patients on antidepressant medication excluded)	Non-healing surgical wound in 90 days	Patients with depressive disorders had higher incidence and odds of non-healing surgical wounds, 0.12% vs 0.04%; OR = 2.81, [1.42–5.55], p = 0.002	Strong
Freshman et al., 2021 ^†^ USA	Hip arthroscopy; Retrospective cohort study using Mariner/PearlDiver database between 2010 and 2019	Matched control group: 5,634; 41.0 ± 13.4; 80.1% Depression group: 5,814; 41.0 ± 13.4; 80.1%	Depression diagnosis in the year before surgery	Superficial SSI up to 90 days post operation	Depressed patients had higher rates of infection (4.9%) compared to non-depressed patients (2.8%), OR = 1.76, [1.44, 2.14], p < .001	Strong
Gold et al., 2020 ^†^ USA	Primary TKA; Retrospective cohort study using an institutional database (15 hospitals) between 2017 and 2019	Control group: 9,350; 67.9 ± 13.4; NR Depression group: 1,396; 66.2 ± 9.6; NR	Depression diagnosis recorded	-Wound complications -PJI	Compared to patients without an identified psychiatric diagnosis, depressed patients did not have higher odds of -wound complications, OR = 0.807 [0.317, 2.053), p = .653, or -PJI OR = 0.762 [0.366–1.589), p = .469,	Strong
Pan et al., 2019 USA	Primary TKA; Retrospective cohort study using the National Inpatient Sample database between 2002 and 2014	Control group: 6,312,129; 66.69 ± 19.23; 61.05% Anxiety group: 35,589; 62.32 ± 23.58; 68.23% Depression group: 788,794; 63.57 ± 22.66; 79.92%	Anxiety or depression diagnosis	Wound dehiscence	Rates of wound dehiscence not significantly different in patients with no psychiatric diagnoses (0.07%) compared to patients with diagnoses of anxiety (0.11%), depression (0.1%), or anxiety and depression (0.2%), p values NR.	Strong
Schwartz et al., 2020 ^†^ USA	Primary THA; Retrospective cohort study using the Truven MarketScan Database between 2009 and 2018	Control group: 158,427; age 18 + ; 49.1% Depression (no psychotherapy) group: 10,912; age 18 + ; 30.2%	Depression diagnosis in the year before surgery	Up to 90 days post operation, rates of -wound complications; -superficial SSI; -deep PJI.	Compared to non-depressed patients, depressed patients did not significantly differ on rates of: -wound complications OR = 0.98, [0.85, 1.15], p = .841; -surgical site infection OR = 0.93, [0.78, 1.11], p = .405; -PJI OR = 1.17, [0.99, 1.39], p = .056.	Strong
Zalikha et al., 2021 ^†^ USA	THA or TKA; Retrospective cohort study using the National Inpatient Sample database between 2006 and 2015	Control group: 7,618,986; 66.55, SE = 0.04; 58.32% Anxiety group: 60,367; 63.08 SE = 0.10; 65.74% Depression group: 1,031,277; 63.95, SE = 0.04; 77.48%	Anxiety or depression diagnosis	Wound dehiscence	The anxiety group did not have significantly odds of wound dehiscence (0.12%), compared to the control group (0.09%), OR = 1.45 [0.87, 2.42], p = .157, Depressed patients had significantly higher odds of experiencing wound dehiscence, (0.10%) compared with the control group (0.09%), OR = 1.21, [1.05, 1.40], p = .009	Strong
**Shoulder Arthroplasty**						
Lunati et al., 2021 ^†^ USA	Primary TSA; Retrospective cohort study using the Truven MarketScan Database between 2009 and 2017	Control group: 19,414; most common age range: 55–64 years; 47.9% Depression group: 3,209; most common age range: 55–64 years; 67.6%	Depression diagnosis in the year before surgery	-Wound complications up to 90 days post-surgery -PJI up to 1 year post surgery	Patients with depression had higher odds of -wound complications OR = 1.84, [1.20, 2.79] p = .004; and - PJI, OR = 1.41, [1.04–1.90], p = .025.	Strong
Mollon et al., 2016 ^†^ USA	Elective TSA; Retrospective cohort study using the Nationwide Inpatient Sample between 2002 and 2012	No history of depression group: 196,096; 69.5 ± 10.0; 53.02% History of depression group: 27,964; 67.3 ± 9.6; 71.25%	History of clinical depression recorded	-Wound haematoma or seroma -Wound dehiscence	No significant differences between the history of depression group and the non-depressed group in rates of: -wound dehiscence (0.04% vs 0.02% respectively, p = .653). -wound haematoma or seroma (0.39% vs 0.38% respectively, p = .908);	Moderate
**Spinal surgery**						
Elsamadicy et al., 2017 ^†^ USA	Elective spine surgery; Retrospective chart review at a single medical centre between 2005 and 2015	Control group: 668; 61.00 ± 15.84; 63.47% Depression group: 255; 61.87 ± 11.59; 65.10%	Psychiatrist diagnosed depression prior to surgery	-Deep SSI -Superficial SSI	No statistically significant difference between non-depressed and depressed patients in rates of: -deep SSI (0.60% vs 1.57% respectively, p = .23); -superficial SSI (0.75% vs 1.18% respectively, p = .69).	Moderate
Menendez et al., 2014 ^†^ USA	Primary spinal fusion or laminectomy; Retrospective cohort study using National Hospital Discharge Survey database between 1990 and 2007	Control group: 4,951,756; 54 ± 15; 49% Anxiety group: 134,559; 53 ± 13; 68% Depression group: 242,205; 53 ± 13; 67%	Anxiety or depression diagnosis	Wound complications	Wound complication rate were: -1.9% in depression group; -2% in anxiety group; -and 1.7% in patients with no psychiatric condition. (Statistical comparison of these groups NR)	Moderate
Wang et al., 2023 ^†^ China	Short segment fusion for degenerative lumbar spinal disease; Retrospective single centre study using database collected between 2018 and 2020	Control group: 201; 79.8 ± 3.4; 56.7% Depression group: 30; 79.3 ± 3.5; 60.0%	Depression measured SDS (scores > 50 classified as depressed	SSI	1/20 depressed patients had surgical site infection, while 4/201 non depressed patients had surgical site infection. (statistical testing NR)	Moderate
**Dental procedures**						
George et al., 1980 ^†^ USA	Molar removal; Prospective study at a single site with 2-week post-surgery follow up	38; age range: 17–32; 52.63%	-Anxiety about recovery: Two question rating scale regarding concerns about recovery/complications -Trait anxiety: Two question rating scale about how tense/relaxed or easily upset they generally were -Psychological assessments administered immediately prior to surgery	Healing rated by experimenter on a seven-point scale ranging from poor to excellent	Healing ratings were not correlated with trait anxiety (0.12) or anxiety about recovery (0.06) (Type of correlation statistic and p values NR)	Weak
Kloostra et al., 2006 ^†^ USA	Periodontal treatment (surgical and non-surgical); Prospective study at a single site with 2-week post-surgery follow up	70; 54.78 ± 13.21; 51.43%	-Trait anxiety measured using the STAI -Depression measured using short version of the CES-D -Both administered before surgery	Two weeks post treatment dentists rated patients’ level of wound healing, outlining: -level of wound healing (primary, secondary, or tertiary); -wound epithelialization (complete and incomplete); -wound integrity (no tissue sloughing, minor tissue sloughing at wound edge only, and major tissue sloughing).	There was a statistically significant negative correlation between trait anxiety and level of wound healing (r = -.316; p = .047). No significant correlations between trait anxiety and -wound epithelialization (r = -.214; p = NR); -and wound integrity (r = -.226; p = NR). No significant correlations between depression and: -level of wound healing (r = -.256, p = .094); -wound epithelialization (r = -.294, p -.053); -wound integrity (r = -.288, p = .064)	Moderate
Yahya et al., 2021 ^†^ Israel	Wisdom teeth extraction; Prospective study at a single site with 6-week post-surgery follow up	94; 45.5 ± 20.60; 3.32%	Preoperative anxiety using a 10 cm visual analogue scale at time of surgery (before anaesthetic)	Healing assessed using the novel Inflammatory Proliferative Remodelling Scale. Administered at three points: 1) Inflammatory phase: 3 − 5 days post extraction; 2) Proliferative phase: 14 days post extraction; 3) Remodelling phase: 6 weeks post extraction.	Non-significant negative correlations between the mean preoperative anxiety healing at each phase: -inflammatory phase: r = -.12, p = .34; -proliferative phase: r = -.02, p = .86; -remodelling phase: r = -.13, p = .64.	Weak
**CABG**						
Beresnevaitė et al., 2010 ^†^ Lithuania	CABG; Prospective study looking at complications during hospital stay (follow up time NR) in a single site	109; 57.8 ± 6.7; 0%	Depression measured using SCL-90R one day before surgery	Recorded occurrence of leg wound infection	Each increase in depression score associated with almost doubled the odds of leg wound infection OR = 1.99, [0.99, 1.46], p = .066. Leg wound infection occurred in 8% of patients with high depression (SCL-90R score 71+) compared with 1.2% of patients with low depression (SCL-90R score <71).	Moderate
Doering et al., 2008 USA	CABG; Prospective study at two sites with 6 month follow up	Control group: 39; 64.3 ± 10.3; 100% Minor depression group: 15; 54.5 ± 18.2; 100% Major depression group: 13; 60.7 ± 8.5; 100%	-Depression was assessed post-surgery, prior to discharge using structured clinical interview (DISH). -Patients were classified as having no depression, minor depression or major depression.	Rates of wound infections for 6 months post-surgery were identified by patient report using the Modified Health Review and by medical chart audit.	No statistically significant difference in rates of wound infections between patients with no depression, minor depression or major depression. (Numerical statistical outcomes NR).	Moderate
Doering et al., 2005 ^†^ USA	CABG; Prospective study at a single site with 6-week post-surgery follow up	Lower depressive symptoms group: 36; 68.1 ± 10.1; 41.7% Higher depressive symptoms group: 31; 60.2 ± 8.9; 32.3%	-Depression at the time of hospital discharge measured with MAACL -Median split was used to divide into groups with high/low depression.	Recorded wound complications	Patients with higher depressive scores had significantly higher rates of wound complications compared to the group with lower depressive symptoms (46% vs 19%, respectively), OR = 3.71, [1.15, 12.0], p = .03	Moderate
Scheier et al., 1999 USA	CABG; Prospective study at single site with 6 month follow up	283; 62.8 ± 10.4; 30.1%	-Neuroticism was assessed with a 10-item version of the Neuroticism scale of the Eysenck Personality Questionnaire. -Depression was assessed with a 10-item version of the CES-D. -Psychological assessments were completed 1–20 days prior to surgery.	Rehospitalizations caused by postsurgical sternal wound infection as ascertained by patient and physician report at 6 month follow up	Neuroticism was not significantly related to rates of hospitalisation for sternal wound infection, b = .31, SE = .19, p = NR Depressed patients were more likely to be re-hospitalised for sternal wound infection than less depressed patients, b = .24, SE = 09; p < .01; n = 254; OR = 5.38, [1.67, 17.37]	Moderate
Tyerman et al., 2021 ^†^ USA	Cardiac operations; Retrospective review study using Society of Thoracic Surgeons database between 2002 and 2017	Control group: 15,339; 66 ± NR; 30.1% Anxiety group: 97; 51 ± NR; 53.6% Depression group: 1,148; 61 ± NR; 45.4%	Anxiety or depressive (mood) disorder diagnoses recorded preoperatively or 30 days post operatively	Deep sternal wound infection	No statistically significant difference in rates of deep sternal wound infection in patients with anxiety (0/97, 0%), or depressive disorder (0/1148, 0%) compared to patients with no serious mental illness (4/15,336, 0.04%)	Moderate
**Mixed surgeries**						
Britteon et al., 2017 England	Mixed common surgeries (hip replacements, knee replacements, hernia repairs, varicose vein operations); Retrospective study using NHS Patient Reported Outcome Measures programme between 2009 and 2011	178,622; age 12 + ; NR	Patients’ presurgical report indicating whether they had received a depression diagnosis	- Patient reported wound complications recorded in PROMS questionnaire. - Hospital reported wound complications or wound related readmissions in clinical notes within 3- or 6- months post-surgery.	Previously diagnosed depression was not significantly associated with hospital-reported wound complications, OR = 0 ⋅ 96, [0 ⋅ 69, 1 ⋅ 33], but, was significantly associated with readmission for a surgical wound complication, OR = 1 ⋅ 37, [1 ⋅ 11, 1 ⋅ 69]	Strong
Broadbent et al., 2003 New Zealand	Inguinal hernia elective surgery; Prospective study at single site tracking cytokine response up to 20 hours post-surgery	47; 63.36 ± 16.42; 12.76%	Preoperative worry about operation was assessed using a 10 cm visual analogue scale from ranging from “not at all worried” to “extremely worried”	Levels of interleukin-1, interleukin-6, and matrix metalloproteinase-9 in the wound fluid	Greater worry about surgery predicted lower levels of metalloproteinase-9 (β = −.38, p = .03), but did not predict levels of interleukin-1 (β = -.15, p = .39) or interleukin-6 (p > 0.5, further data NR).	Moderate
Drinane et al., 2019 ^†^ USA	Breast reconstruction after mastectomy; Retrospective cohort study using National Inpatient Sample between 2010 and 2013	Control group: 157,454; 51.27 ± 10.67; 99.8% Depression group: 17,957; 52.32 ± 9.93; 99.8%	Documented diagnosis of depression	Documented wound complications	Depressed patients had higher rates of wound complications (1.8%) compared to non-depressed patients (1.2%), adjusted OR = 1.6, [1.41, 1.8], p < .001	Strong
Jovanovic et al., 2022 Serbia	Vascular surgery; Single site cross sectional study	385; 67.1 ± NR; 20.8%	Preoperative anxiety (anaesthesia and surgery related anxiety) assessed with Serbian version of APAIS. Patients with diagnosed anxiety disorder excluded.	Local wound infection	Local wound infection was not associated with anaesthesia related anxiety (r = .001, p = 0.991) or surgery related anxiety (r = 0.049, p = 0.341) ^*^	Moderate
Kassahun et al., 2022 Germany	Major general surgery Single site prospective observational study	Control group: 159; 59.28 ± 13.31; 35.2% Anxiety group: 241; 57.90 ± 14.65; 53.5%	Preoperative anxiety measured using the STAI (STAI <40 no anxiety; STAI ≥40 anxiety)	SSI	No significant difference in rates of SS in anxious patients (14.5%) compared to control group (13.2%), p = 0.711	Moderate
Oduyale et al., 2021 ^†^ USA	Colectomy or proctectomy; Retrospective cohort study using National Inpatient Sample between 2002 and 2017	Colectomy patients -Control group: 3,356,487; 62.73 ± 17.04; 52.41% -Depression group: 241,916; 62.48 ± 14.91; 69.74% Proctectomy patients -Control group: 573,574; 60.81 ± 18.06; 50.58% -Depression group: 40,148; 61.47 ± 15.02; 70.14%	Documented diagnosis of preoperative depression	Documented wound infection	Depression was associated with higher risk of wound infection in both colectomy, OR = 1.08, [1.03, 1.12], p < .001 and proctectomy patients, OR = 1.19, [1.05, 1.35], p = .006	Strong
Pedras et al., 2022 ^†^ Portugal	Lower extremity amputation due to diabetic foot ulcer; Prospective study at six major hospitals with 10 month follow up	149; 65.5 ± 10.7; 29.5%	Anxiety and depression measured using the HADS	Time to lesion healing (Classification as healed at 1, 6, or 10 month follow up)	Preoperative anxiety predicted healing HR = 0.96 [0.95, 0.97], p < 0.05. Healing was negatively associated with anxiety and month 1 (r = -0.186, p < 0.05) and month 10 (r = -0.315, p < 0.01) Depression was not associated with healing, HR = 0.97, [0.92, 1.004].	Strong
Zhang et al., 2021 ^†^ USA	Colectomy; Retrospective cohort study using Marketscan database between 2010 and 2017	Control group: 67,103; age range: 18–64; 45% Depression group: 21 878; age range: 18–64; 65.62%	History of depression within the past year identified from recorded diagnoses or antidepressant use.	Documented wound infection	Rates of wound infection were not significantly different in patients with a history of depression (3.26%) compared to patients with no history of depression (3.11%, p = .245), adjusted OR = 1.05, [0.96, 1.15], p = .241	Strong

*Note*.

† indicates study is included in meta-analysis.

NR = not reported.

Surgery types: CABG = Coronary Artery Bypass Graft; TAA = Total Ankle Arthroplasty; THA = Total Hip Arthroplasty; TKA = Total Knee Arthroplasty; TSA = Total Shoulder Arthroplasty

Wound outcomes: PJI = Prosthetic Joint Infection; SSI = surgical site infection

Anxiety or depression measures: APAIS = Amsterdam Preoperative Anxiety and Information Scale [80]; CES-D = Center for Epidemiologic Studies Depression Scale [81]; DISH = The Depression Interview and Structured Hamilton [82]; Eysenck Personality Questionnaire [83]; HADS = Hospital Anxiety and Depression Scale [84]; MAACL = Multiple Affect Adjective Check List [85]; SCL-90R = 90-item Symptom Checklist Revised [86]; SDS = Self-rating Depression Scale [87]; STAI = State-Trait Anxiety Inventory [88]

* Data obtained following correspondence with lead author.

**Table 2:** Characteristics and results of studies of ulcer and burn wounds.

Authors, year Country	Study design characteristics; Context; Country	Participants: N; age ± SD; % female	Anxiety or depression measure	Ulcer/burn characteristics Wound healing outcome measure	Findings	Quality rating
**Ulcers**						
Bui et al., 2017 Australia	Cross-sectional study; Outpatient clinics and community settings	561; 71.16 ± 14.41; 49.90%	Depression diagnosis	-Chronic leg ulcers (4 + weeks duration) -Ulcer infection clinically diagnosed and documented	Being diagnosed with depression was associated with a significantly higher risk of ulcer infection, β = 1.02, OR = 2.27, 95%CI [1.08, 7.19], p = .035	Moderate
Bui et al., 2018 Australia	Prospective study with 12 week follow up; Outpatient clinics and community settings	636; 71.1 ± 14.35; 50.0%	Diagnosis of depression (patient report and review of medical records)	-Chronic leg ulcers (4 + weeks duration) -Development of ulcer infection clinically diagnosed and documented. (No infection at baseline).	Depression diagnosis not significantly associated with increased risk for infection, β = 0.58, HR = 1.79, [0.97, 3.28], p = .062	Moderate
Chaby et al., 2013 France	Prospective study 24 week follow up; Dermatology departments (n = 22)	104; 73.5 ± 9.9; 71%	Depression measured using BDI	- Venous leg ulcers (4 + weeks duration and with wound area > 1 cm2) -Wound classified as healed or non-healed at 24 weeks	Depression scores were similar across the healed and non-healed groups (significance tests NR)	Moderate
Cole-King et al., 2001 UK	Cross sectional study; Wound Healing Research Unit	53; age range 22–91; 58.49%	-Anxiety and depression measured using the HADS	-Chronic leg ulcers -Wound healing was rated using a five-point Likert scale. -Rating was informed by clinical judgement and tracking of wound area. -Patients grouped into: healing well (scores 1/2) or delayed healing (scores 3/4/5).	Anxiety -Anxiety caseness (HADS anxiety 9+) was associated with delayed healing, Mann-Whitney test: Z = 1.9806, p = .0476 -Fifteen out of the 16 patients scoring 9 + HADS anxiety had delayed healing (Fisher exact test, p = .02625 Depression -Depression caseness (HADS depression 9+) was associated with delayed healing, Mann-Whitney test: Z = 2.1560, p = =.0311. -All 13 patients classified scoring 9 + HADS depression had delayed healing (Fisher exact test: p = .00965).	Weak
Finlayson et al., 2014 ^†^ Australia	Randomised control trial (comparing two compression bandage systems); Outpatient clinics in hospital and community settings	103; 68 ± 14.8; 50%	Depression measured using GDS	- Venous leg ulcer 1)Rate of healing was assessed every 2 weeks from baseline to week 24 and was tracked using the following metrics: a) ulcer area, b) Pressure Ulcer Scale for Healing tool, c) clinical data, e.g., presence of inflammation, oedema etc. 2) Ulcer healing at 24 weeks - A ‘healed’ leg ulcer was defined as full epithelialisation of the wound, which was maintained for 2 weeks.	Participants scoring >4 on the GDS: -had significantly slower rate of healing (p = .012) -were significantly less likely to heal by week 24 (β = -0.762, HR = 0.47, 95%CI = 0.23–0.96, p = .037) ^**^	Strong
Jess et al., 1989 Denmark	Prospective study with 6 week follow up; Hospital outpatient clinic	56; age range 23–81; 50%	Neuroticism and anxiety index (actual anxiety) measured with MMPI at baseline	- Duodenal ulcer -Participants were classed as either: a) Rapidly healing if healed within 2 weeks (n = 14); b) Slowly healing if healed within 6 weeks (n = 14); or c) Non healing if they had not healed in 6 weeks (n = 28).	A statistically significant trend was found, indicating that a high anxiety index was not in favour of spontaneous ulcer healing (p < 0.05, Jonckheere-Terpstra test for trend) but no other significant differences between the groups.	Moderate
Levenstein et al., 1996 Italy	Prospective study with 6 week follow up; NR	70; 41.7 ± 14; 24%	Anxiety: Unified anxiety score created using STAI and Self-Administered Anxiety Scale (version of Zung’s SAS) Depression: Unified depression score created using CES-D and Self-Administered Depression Scale (version of Zung’s SDS)	-Duodenal ulcer -Endoscopy determined whether A) ulcer was closed or persistent (not closed) B) tissue completely healed (normalization of the duodenal mucosa) or incompletely healed	Anxiety associated with increased risk for -persistent ulcer (OR = 1.04 per increase of 0.01 in anxiety score, CI = NR, p = .04) -incomplete healing (OR = 1.03, CI = NR, p = .03). Depression not significantly associated with -risk of persistent ulcer (p = .13) -incomplete healing (p = .07; ORs NR).	Moderate
Melikian et al., 2019 USA	Retrospective cohort study; Academic vascular and wound centre	65; 60 ± NR; 41.67%	Documented depression (no further information)	-Venous leg ulcer -Classification as healed or unhealed after a minimum of 52 weeks treatment	After adjusting for demographic variables, depression was not associated with ulcer healing, OR 3.73, [0.21, 66.99], p = .37	Moderate
Monami et al., 2008 ^†^ Italy	Prospective study with 6 month follow up; Type 2 diabetic patients referred to geriatric unit	80; 74.4 ± 8.3; 56.3%	Depression measured using GDS	- Chronic diabetic foot ulcer (duration > 3 months) -Classification as healed or unhealed after 6 months	Patients who healed had significantly lower depressive symptoms compared to non-healers (6.0 ± 4.2 versus 16.1 ± 6.1; p = .005). Those with high depressive scores (≥ 10) had a significantly higher risk of not healing at 6 months, HR = 2.004, [1.131, 3.542], p = .017	Moderate
Onoyama et al., 2020 Japan	Prospective study with 6 month follow up; Department of Vascular Surgery at local hospital	50; 69.2 ± 9.8; NR	Depression measured using GDS	-Peripheral arterial disease limb lesions/ulceration -The rate of requiring local medical treatment for the lesions within 6 months after the initial treatment or hospitalization was taken to indicate poor healing. This outcome was identified in medical records.	Depression was not related to local medical treatment (significance testing results NR).	Weak
Takahashi et al., 2009 ^†^ USA	Retrospective chart review; Long term care residents referred to a wound consultative service	397; 78.1 ± 11.25; 53%	Depression identified in medical notes	- Pressure, ischemic, venous, neuropathic, or mixed ulcer -Classification as healed or not healed. -Complete healing was defined as 100% closure of the wound within 6 months.	After adjusting for demographic variables, depression was not significantly associated with ulcer healing OR = 0.70, [0.37, 1.34]	Moderate
Udovichenko et al., 2017 Russia	Prospective study with 1.5 year follow up; Diabetic foot outpatient clinic,	285; 65 (range 25–91); 55%	Anxiety measured using HADS anxiety Depression measured using CES-D	- Diabetic foot ulcer and leg ulcers - Time to ulcer healing in days	No difference in time until healing for patients with anxiety (scoring 8+) versus no anxiety (data NR). No significant difference between in median days until healing in patients with depression (scoring 18 + on CES-D; median days: 156), and patients without depression (median days: 155), p = non-significant (p value NR)	Moderate
Vedhara et al., 2010 ^†^ UK	Prospective study with 24 week follow up; Outpatient podiatry clinics in secondary care	93; 60.7 ± 10.97; 26.88%	Anxiety and depression measured using the HADS	- Diabetic foot ulcer - Ulcer assessments involved determining over the 24 week follow up (1) whether the ulcer had healed (2) changes in ulcer size, i.e., ulcer area measured at 0, 6, 12 and 24 weeks	Anxiety did not significantly predict -whether ulcers healed during study period, OR 2.293, [0.509, 10.324], p = .280, -or change in ulcer area, (F = 1.297, p = .281, d = 0.115) -Depression did not significantly predict whether ulcer healed during study period, OR 2.389, [0.358, 15.392], p = .368 -Patients with depression (HADS depression score 11+) had significantly smaller changes in ulcer size over time, F = 5.30, p = .004, d = 0.31.	Moderate
Walburn et al., 2017 UK	Prospective study with 24 week follow up; Primary care leg ulcer outpatients clinics	63; 68.1 ± 15.8; 60.3%	Anxiety and depression measured using the HADS	- Venous leg ulcer - Rate of change in ulcer surface area measured at weeks 0, 6, 12, 24. (using VISITRAK method) - Time from baseline until the ulcer healed, up to week 24 (using nurses judgement)	Anxiety did not significantly predict -change in ulcer area per week, b = -0.01, SE = 0.016, β = -0.18, p = .53 -or time until the ulcer was deemed healed, HR = 0.987, [0.833, 1.104], p = .88) Depression significantly predicted change in ulcer area per week, b = -0.033, SE = 0.016, β= -0.514, p = .039, but not time until the ulcer was deemed healed, HR = 1.003, [0.869, 1.157], p = .97	Strong
**Burns**						
Wilson et al., 2011 UK	Prospective study with follow up duration of weeks (n weeks not specified); Outpatient clinic of a regional burns service	72; 43.64 ± 16.4; 54%	-Anxiety and depression measured using the HADS -HADS administered shortly after burn injury	- Mixed burns - Wound healing time defined as number of days between injury and being declared healed in medical notes or being referred to scar management clinic.	No associations between anxiety and burn healing time (data NR). HADS depression score was positively correlated with log burn healing time (Spearman’s rho = .280, p = .028).	Moderate
Tarrier et al., 2005 UK	Retrospective case controlled study; Inpatient burns unit	Matched control group: 15; 51.0 ± 19.8; 100% Depression group: 8; 50.8 ± 16.2; 100%	Pre burn depressive disorder identified in medical notes	- Mixed burns - Amount of time between injury and healing in weeks. Healing was recorded in medical notes when the surface of the wound had reepithelialised and new skin was present.	Patients with depression did not differ significantly from their matched controls on average time (in weeks) taken for wound to heal (19.3 ± 18.1 vs 9.7 ± 7.4 respectively, p = NR).	Moderate

*Note*.

† indicates study is included in meta-analysis.

NR = Not reported

** Although the marked study involve intervention groups, the reported analyses is carried out on the total study sample.

Anxiety or depression measures: BDI = Beck Depression Inventory [89]; CES-D = Centre for Epidemiologic Studies Depression Scale [81]; GDS = Geriatric Depression Scale [90]; HADS = Hospital Anxiety and Depression Scale [84]; MMPI = Minnesota Multiphasic Personality Inventory [91]; SAS = Self-rating Anxiety Scale [92]; SDS = Self-rating Depression Scale [87]; STAI = State-Trait Anxiety Inventory [88]

**Table 3:** Characteristics and results of studies of experimental wounds.

Authors, year Country	Characteristics of study design	Participants n sample description; Mean age ± SD; % female	Anxiety or depression measure	-Wound type -Wound healing outcome measure(s)	Key findings	Quality rating
Bosch et al., 2007 ^†^ USA	Students with high or low loneliness or depressive symptoms were wounded and monitored daily until healed.	193 undergraduate students; 20.1 age range = 18–31; 50.78%	Depression measured using BDI-SF	-3.5mm circular wound on the oral hard palate -Wound size measured daily by two independent raters. -Wound classified as healed when closure exceeded 95%.	Depressive symptoms (continuous BDI-SF scores): -had a significant effect on wound size F(8,166) = 2.56; p = .01 -were significantly associated with time needed to heal, OR per SD on the BDI-sf 1.50, 95% CI [1.10, 2.06], p = .007 Incidence of depression (scoring 8 + on BDI-SF, n = 40): -had a significant effect on wound size, F(8,166) = 2.51; p = .01 -was associated with a slower than median healing rate, adjusted OR = 3.68, [1.37, 9.89], p < .05	Moderate
Glaser et al., 1999 USA	Proinflammatory cytokines at wound site were tracked for up to 24 hours.	36 postmenopausal women; 57.2 ± 6.6; 100%	Negative affect measured using PANAS	-Eight 8mm suction blisters on forearm -Levels of interleukin-1 alpha and interleukin -8 in blister chamber wound fluid. -Median splits applied to both cytokine levels.	Negative affect was higher in subjects who had low levels of both cytokines after 24 hours (n = 13, mean negative affect score 21.0 ± 8.5) compared to those who had high cytokine levels (n = 15, mean negative affect score = 15.1 ± 4.7), F(1, 27) = 5.26, p = .03	Weak
Gouin et al., 2008 USA	-Participants randomised into a relaxation training or control group. -Following wounding and on the same day, the relaxation group completed three relaxation sessions. -Healing tracked daily for 8 days.	98 healthy volunteers; most common age range: 30–49 years; 59.2%	-Anxiety measured using BAI -Depression measured using BDI-SF -Negative affect measured using PANAS	-Eight 8mm suction blisters on forearm -Healing assessed by rate of trans epidermal water loss -Median split used to distinguish “ fast healers” (</ = 4 days) from “ slow healers” (>4 days)	Neither anxiety, depression, or negative affect were significant predictors of healing status at day 4 (Wald statistic; data NR) ^**^	Moderate
Koschwanez et al., 2013 New Zealand	-Participants randomised to write for 20 minutes a day about either upsetting life events (Expressive Writing) or about daily activities (Time Management) for 3 consecutive days. -Two weeks post writing wounds experimental wounds created and monitored for 3 weeks.	49 healthy older volunteers; 78.8 ± 7.2; 57.1%	-Depression measured using GDS	-4-mm punch biopsy wounds on the inner, upper arm -Wounds were photographed every three to five days for 21 days to monitor healing. -Classified as healed when achieved full reepithelialisation as determined by a dermatologist.	Depressive symptoms did not significantly predict rate of wound healing (data NR). ^**^	Moderate
McGuire et al., 2006 USA	-Prior to surgery on surgery, on day of surgery, wound was created. -Healing was monitored up to 4 weeks.	17 gastric bypass surgery patients; 37.65 ± 7.79; 100%	Depression measured using BDI-SF	-2-mm punch biopsy on the back of upper arm -Wound size assessed using digital photograph at days 1, 2, 7, 10, 14, 17, 21, 24, and 28 post wounding. -Measurement completed by two independent raters aided by software programme.	No significant difference in time to healing between participants with no depressive symptoms (BDI-SF score < 5, n = 6) and those with depressive symptoms (BDI-SF score 5 + , n = 7), log-rank test, p = .54	Moderate
Robles et al., 2013 USA	-Participants visited laboratory twice with almost identical procedures. -Skin barrier function was disrupted using a tape-stripping procedure. -This was followed by a 20-minute discussion of personal concerns or relationship problems (one topic per visit). -Skin barrier recovery was assessed up to 2 hours after skin disruption.	68 healthy dating couples (34 couples); 22.43 ± 3.88; 54.68%	Attachment anxiety measured using the ECR-R	-Tape-stripping on arm -Recovery measured using trans epidermal water loss at hours 1, 1.5, and 2 post skin -disruption.	-Attachment anxiety related to *faster* skin barrier recovery across both visits in women (linear estimate = 0.13, SE = 0.04, df = 324, t = 3.47, p.006), but not in men (linear estimate = -0.11, SE = 0.06, df = 337, t = -1.9, p.06). -Attachment anxiety was related to slower recovery in men during the personal concern discussion only	Weak
Robles et al., 2009 USA	-Skin barrier function was disrupted using a tape-stripping procedure. -Participants were randomly assigned were randomly assigned of the following tasks: No Stress (reading task), Stress (Trier Social Stress Test), or Stress + Support (Trier Social Stress Test with a supportive confederate). -Skin barrier recovery was assessed up to 2 hours after skin disruption.	60 healthy volunteers; 22.7 ± 3.9; 45%	Negative affect measured using PANAS	-Tape-stripping on arm -Recovery measured using trans-epidermal water loss up to 2 hours after skin disruption	Negative affect did not predict skin barrier recovery (βlinear = 2.03, SE = 4.21, t = 2.95, effect size r = .07) ^**^	Weak
Wilson et al., 2017 USA	-Couples were wounded and engaged in marital problem discussion in the laboratory. -Self report mood and biomarkers assessed repeatedly. -Healing tracked over 32 days.	84 healthy heterosexual married couples (42 couples); 37.0 ± 13.0; 50%	Negative affect measured using PANAS	-2mm dermal punch biopsy wound on arm -Healing measured using trans-epidermal water loss at the wound site on 14 measurement occasions (baseline during visit, then days 1–8, 12, 16, 20, 24, 28, and 32).	Negative affect at baseline did not predict wound healing, β = -0.131, SE = 0.130, [-0.392, 0.13], p = .318	Weak
Yang et al., 2002 USA	-Participants’ skin was exposed to UVB radiation and wounds created in both irridated and non irridated areas. -Wound fluid extracted and analysed 18 hours post wounding.	51 healthy volunteers; 41.72 SEM = 2.42; 60.78%	Depression measured using BDI	-Four 8-mm suction blister wounds on forearms -Levels of tissue inhibitors of metalloproteinases -1, or Matrix metalloproteinases -2,-8 or -9, in blister chamber wound fluid	Depressive symptoms were not related to the expression of either matrix metalloproteinases or tissue inhibitors of metalloproteinases in blister chamber wound fluid (data NR)	Weak

*Note*.

† indicates study is included in meta-analysis.

NR = Not reported

** Although the marked study involve intervention groups, the reported analyses is carried out on the total study sample.

Anxiety or depression measures: BAI = Beck Anxiety Inventory [93]; BDI = Beck Depression Inventory [89]; BDI-SF = Beck Depression Inventory – Short Form [94]; ECR-R = Experiences in Close Relationships - Revised [95]; GDS = Geriatric Depression Scale [90]; PANAS = Positive and Negative Affect Schedule [96]

### Quality appraisal

Overall results of the quality appraisal are displayed in Tables 1–3. Eighteen of the included studies were rated as strong, 26 as moderate and 11 were rated as weak. Over half (16 out of 26) of the studies on surgical wounds were rated as strong. This is because several of them utilised population-based cohort study designs, meaning that selection bias was likely to be low and data collection methods were largely deemed as valid and reliable (see Table 1). More than half of the studies on experimental wounds (five out of nine) were rated as weak, this is largely because participants self-referred to these studies, meaning that selection bias may have been an issue and because there was not extensive control of confounding variables (see Table 3). Full quality ratings for each of the components of the appraisal tool are displayed in S3 Table.

### Anxiety and wound healing: Narrative synthesis

Twenty-one of the included studies examined associations between anxiety and healing in various types of surgical, clinical, and experimental wounds [22,25,27,37,39,43,45–47,51,52,54,56,57,59,64,67,68,70,72,79]. The studies employed a range of study designs including retrospective cohort, prospective, cross-sectional and RCT designs. The studies utilised various measures of anxiety including the presence or absence of a diagnosed anxiety disorder as recorded in clinical notes [37,39,43,52], trait anxiety measures [45,46] or validated anxiety symptom measures [22,27,56,57,59,64,67,70,72]. Other studies devised or adapted anxiety measures for the study [45,68]. Two studies utilised visual analogue scales to quantify worry/anxiety prior to surgery [47,54]. Two studies examined the impact of neuroticism [25,51], while one study explored the effect of attachment anxiety, which was described by the researchers as the degree to which individuals worry about rejection or loss of closeness in a romantic relationship [79].

#### Associations between anxiety and rate of wound healing.

Eight studies examined whether there was an association between anxiety and time for (ulcer, burn or surgical) wounds to heal, with mixed results [22,25,59,64,67,68,70,72]. The majority of these studies were rated as moderate (k = 5) in the quality appraisal, two were rated as strong and one was rated as weak. Four studies found that self-report anxiety was associated with incomplete healing (OR = 1.03; 67), delayed healing (Mann Whitney test Z: 1.98; 63), healing time (r = -0.315; 58) or reduced likelihood of spontaneous healing (p < 0.05, Jonckheere-Terpstra test for trend; 25) of ulcers. In contrast, four studies found that self-report anxiety was not predictive of time required to heal for leg ulcer, foot ulcer or burn wounds [22,67,70,72].

#### Effect of anxiety on change in wound surface area over time.

Two studies evaluated whether anxiety predicted change in ulcer area over time [22,67]. In studies that were rated as moderate and strong respectively, it was found that anxiety did not predict changes in wound surface area for diabetic foot ulcers (F = 1.297, p = .281; [22]) or venous leg ulcers (β = -0.18, p = .53; [67]).

#### Associations between anxiety and clinician rating of healing status.

Three studies, rated as weak to moderate in quality, explored associations between self-report anxiety and clinicians’ assessment of healing from dental surgery/treatment using novel rating scales [45–47]. Kloostra and colleagues [46] found a negative moderate correlation (*r* = -.316) between trait anxiety and wound healing but no other significant correlations were found (correlation coefficients range: -.226 to.12; [45,47]).

#### Anxiety and rates of wound complications including infections.

Seven studies explored the influence of anxiety on wound complications and infections [37,39,43,51,52,56,57].

Four studies, rated as moderate to strong in quality, explored whether anxiety was related to rates of wound complications and related outcomes post-surgery. In one study, rates of wound complications were slightly higher in patients with anxiety (2%) compared to patients with no psychiatric disorder (1.7%) however whether this difference was statistically significant was not reported [43]. In the three other studies anxiety was not associated with significantly higher rates of post-surgical wound dehiscence or re-hospitalisations due to wound infection [37,39,51].

Three studies, rated as moderate, explored whether anxiety was associated with rates of surgical wound infection. They found no association between anxiety and rates of local wound infection [56], infection at the surgical site [57] or deep sternal wound infection [52].

#### Anxiety and rate of recovery of skin function.

Two studies, rated as moderate and weak in quality respectively, examined the rate of skin barrier recovery following experimental wounding/skin disruption, as measured by TEWL [27,79]. Gouin and colleagues [27] found that anxiety, did not predict the rate of healing of suction blisters. While Robles and colleagues [79] found that, across two laboratory visits, attachment anxiety predicted faster skin barrier recovery in women (β = 0.13, *p* = .006), but not in men (β = -0.11, *p* = .06) following skin disruption using tape-stripping.

#### Anxiety and biomarkers of wound healing.

Broadbent and colleagues [54] examined wound healing by tracking cytokine levels in wound fluid post-surgery, in a study rated as moderate in quality. They found that anxiety predicted lower levels of metalloproteinase-9 (β = −.38, p = .03) but did not predict levels of interleukin-1 or interleukin-6.

### Meta-analytic synthesis: Anxiety and wound healing

Five studies were included in two meta-analytic syntheses exploring the associations between anxiety and wound healing [39,43,45–47].

#### Correlations between anxiety and clinicians’ rating of wound healing.

**Fig 2:**
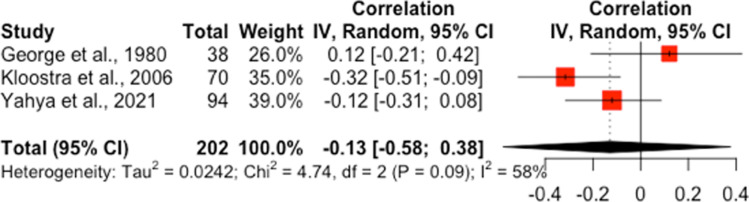
Forest plot of associations between anxiety and clinician’s rating of wound healing.

Three studies, which evaluated the correlations between anxiety and clinician rated healing in dental procedures were synthesised in a meta-analysis. Two studies measured trait anxiety using either a novel two question scale [45] or the State-Trait Anxiety Inventory (STAI; [46, 88]), while one study measured preoperative anxiety using a visual analogue scale [47]. The quality appraisal rated them as weak [45,47] to moderate in quality [46]. The statistical synthesis did not indicate a significant correlation between anxiety and wound healing ratings, *r* = -0.13, 95% CI [-0.58, 0.38], see Fig 2.

## Anxiety and risk of wound complications/dehiscence

**Fig 3:**
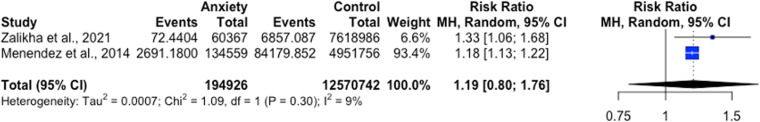
Forest plot of associations between anxiety and rates of wound complications/dehiscence.

Two studies that explored the relationship between a recorded anxiety diagnosis and rates of wound complications following spinal surgery [43] or wound dehiscence following knee or hip arthroplasty [39] were included in a meta-analytic synthesis. The studies were classified as strong [39]and moderate [43] in the quality appraisal. In the pooled analysis, a diagnosis of anxiety did not significantly increase the risk of wound complications/dehiscence RR = 1.19, 95% CI [0.80, 1.76], see Fig 3.

### Depression and wound healing: Narrative synthesis

Forty-eight of the included studies explored associations between depression and wound healing outcomes, utilising a range of wound types including surgical, ulcer, burn and experimental wounds [22,23,26–29,31–44,46,48–53,55,58–74,76–78]; see Tables 1–3). These studies utilised various research designs including: retrospective cohort, prospective, cross-sectional, retrospective chart review, retrospective case controlled and RCTs. Depression was identified/quantified in several ways. Twenty-three studies compared wound healing outcomes in participants with a diagnosis of depression versus participants with no psychiatric diagnosis (e.g., [37,42,53]). Other studies utilised validated measures of depression symptomology (e.g., [37,42,53]), such as the Geriatric Depression Scale (GDS; [90]), the Beck Depression Inventory (BDI; [89]) or the Centre for Epidemiologic Studies Depression Scale (CES-D; [81]). Other studies assessed the level of negative affect using the Positive and Negative affect Schedule (PANAS; [96]; e.g., [73,76]). One study created a novel composite depression measure [68].

#### Association between depression and rate of wound healing.

Seventeen studies explored the relationship between depression and rate of wound healing, with mixed results [22,23,26,28,34,59,63–68,70–72,76,78]). Twelve of these studies were rated as moderate in the quality appraisal, four were rated as strong, and one was rated as weak [64], see Tables 1–3.

Two studies found that depressive symptoms were associated with delayed healing of leg ulcers [64, 65]. However, four studies found no association between depression and healing time for duodenal or foot/leg ulcers, or surgical wounds [59,67,68,70]. In burns patients, Wilson and colleagues [72] found a small positive correlation (ρ = .28) between depressive symptoms and healing time, whereas Tarrier and colleagues [71] found that patients with depression did not differ from matched controls in time taken for burns to heal. In experimentally induced wounds, one study found that individuals with high self-report depressive symptoms were over 3.5 times more likely that those with low depressive symptoms to exhibit slower than median healing rate of a wound on the oral hard palate (OR = 3.68, 95% CI [1.37, 9.89]; [78]). However, two further studies found no association between depressive symptoms and healing time for punch biopsy wounds [28,76].

Six studies examined whether there was an association between depression and whether an ulcer was classified as healed or unhealed by the end of the study follow-up period. Two of these studies found that patients with higher depressive scores had higher risk of leg (HR = 0.47, 95% CI [0.23, 0.96]; [65]) or foot ulcers (HR = 2.00, 95% CI [1.131, 3.542]; [26]) at 6-month follow-up. None of the other four studies found a significant association between depression and ulcer healing in studies with 6-month [22,23,63] or 12-month follow-up [66]. One study found that patients with depressive disorder had higher odds of surgical wounds not being healed by 90 day follow up [34].

#### Depression and change in wound surface area over time.

Three studies, which were rated as moderate [22,78] to strong [67] in quality, explored whether depression was associated with change in wound surface area over time. All three demonstrated an effect of depression: Depression had a small effect on wound size (*F*(8,166) = 2.51; *p* = .01) in an experimental wound model [78] and had a moderate effect on changes in ulcer diabetic foot size over time (d = 0.31; [22]). Furthermore, depression significantly predicted change in wound area per week in venous leg ulcers (β= -0.514, p = .039; [67]).

#### Association between depression and clinician rated healing status.

One study, which was rated as moderate in the quality appraisal, investigated an association between depression and clinician ratings of wound healing following periodontal treatment [46]. Preoperative depression was not significantly associated with dentist rating of wound healing (*r* = -.256, *p* = .094), wound epithelialization (*r* = -.294, *p* = .053), or wound integrity (*r* = -.288, *p* = .064).

#### Depression and wound complications including infections.

Overall, 24 of the included studies examined the association between depression and wound complications [31–33,35–43,48,49–53,55,58–62]. All of the studies related to surgical or ulcer wounds.

Nine studies explored the association between depression and overall wound complication rate post-surgery, all of which were rated as moderate to strong in the quality appraisal. Five studies found that patients with depression had higher odds of wound complications than non-depressed patients following ankle surgery (OR = 1.13; 95% CI [1.00, 1.28]; [31]), total ankle arthroplasty (TAA; OR = 1.59, 95% CI [1.11, 2.29]; [32]), total shoulder arthroplasty (TSA; OR = R = 1.41, 95% CI [1.04–1.90]; [40]), breast reconstruction following mastectomy (OR = 1.6, 95% CI [1.41, 1.8]; [55]), or CABG (OR = 3.71, 95% CI [1.15, 12.0]; [50]). Menendez and colleagues [43] reported that depressed patients had higher rates of wound complications (1.9%) that non-depressed (1.7%) patients but did not report a statistical comparison of the groups. Britteon and colleagues [53] found that previously diagnosed depression was not associated with odds of hospital-reported wound complications (OR = 0.96, 95% CI [0.69, 1.33] but, was associated with higher odds of readmission rates for surgical wound complication (OR = 1.37, 95% CI [1.11, 1.69]). Two studies found that no difference in odds of wound complications between depressed and non-depressed patients following total knee arthroplasty (TKA; OR = 0.807, 95% CI [0.317, 2.053]; [36]) or total hip arthroplasty (THA; OR = 0.98, 95% CI [0.85, 1.15]; [38]).

Four studies, rated as moderate to strong in the quality appraisal, explored rates of wound dehiscence in depressed versus non depressed patients post-surgery, with mixed findings. Zalikha and colleagues [39] found that depressed patients had significantly higher odds of experiencing wound dehiscence compared to patients with no psychiatric diagnosis following THA or TKA (OR = 1.21, 95% CI [1.05, 1.40]. On the other hand, three studies found no difference between depressed and non-depressed patients’ rates of wound dehiscence following TAA (OR = 1.58, 95% CI [0.93, 2.58]; [32]), (TKA (0.1% versus 0.07% respectively; [37]) or TSA (0.04% versus 0.02% respectively, *p* = .908; [40]). Further, Mollon and colleagues [40] found no differences between depressed and non-depressed patients in rates of wound haematoma or seroma following TSA (0.04% vs 0.02% respectively, *p* = .653).

Studies exploring the association between depression and wound infection also generated mixed results and were rated as moderate to strong in the quality appraisal. Diagnosed depression was associated with a higher risk of infection in chronic leg ulcers in a cross-sectional study (β = 1.02, p = .035; [61]), but this finding was not replicated in a subsequent longitudinal study by the same research group (β = 0.58, p = .062; [62]). Depression was associated with a higher risk of wound infection in colectomy (OR = 1.08, 95% CI; [1.03, 1.12]) and proctectomy (OR = 1.19, 95% CI [1.05, 1.35]) patients [58] and patients with high depressive symptoms were more likely to be re-hospitalised for sternal wound infection following CABG (OR = 5.38, 95% CI [1.67, 17.37]; [51]). However, three studies did not find a statistically significant effect of depression on risk of wound infection following CABG (OR = 1.99, 95% CI [0.99, 1.46]; [48,49]) or colectomy (OR = 1.05, 95% CI [0.96, 1.14]; [60]).

In the context infection characteristics, two studies found that rates of superficial surgical site infection (SSI) were higher in depressed patients ([32,35], while three others did not [38,42,44]). Two studies found no impact of depression on rates of deep wound infections [42,53]. The association between a diagnosis of depression and prosthetic joint infections was explored in five studies. Studies led by Bozic [33], Lunati [40] and Wilson [72] found that depressed patients had greater risk/odds of prosthetic joint infections following TKA (HR = 1.28, 95% CI [1.08, 1.51]), TSA (OR = 1.41, 95% CI [1.04, 1.90]), and TAA respectively (OR = 1.82, 95% CI [1.06, 3.15]). On the other hand, two other studies found that depressed patients did not differ from non-depressed patients in rates of prosthetic joint infections following TKA (OR = 0.762, 95% CI [0.366, 1.589]; [36]) or THA (OR = 1.17, 95% CI [0.99, 1.39]; [38]).

One study took a slightly different approach and explored the relationship between depressive symptoms and the rate of requiring local medical treatment for peripheral arterial disease limb lesion wounds [69]. The requirement of additional treatment was within a 6 month period following the initial treatment or hospitalisation and taken to indicate poor wound healing. This study, which was rated as weak in the quality appraisal, found that depression was not related to the rate of requiring medical treatment as identified in medical notes.

#### Depression and rate of recovery of skin function.

Three studies, that were rated weak to moderate in the quality appraisal, utilised the TEWL method to explore associations between depressive symptoms and rate of recovery of skin function. Depression did not predict healing in suction blister wounds [27], punch biopsy wounds (β = -0.131, p = .318; [77]), nor tape stripping (β = 2.03, SE = 4.21; [29]).

#### Depression and biomarkers of wound healing.

Two studies explored the relationship between depressive symptoms and biomarkers of wound healing in experimentally created suction blister wounds [73,74]. Both studies were rated as weak in the quality appraisal. Yang and colleagues [74]) found that depressive symptoms were not related to the expression of healing biomarkers in wound fluid. On the other hand, Glaser and colleagues [73] found that negative affect was higher in subjects who had low levels of interleukin-1 alpha and interleukin -8 in blister chamber wound fluid, compared to those with high levels of these proinflammatory cytokines (F(1, 27) = 5.26, p = .03).

### Meta-analytic synthesis: Depression and wound healing

Twenty-three studies were included in four meta-analytic syntheses investigating the relationship between depression and wound healing [22,23,26,31,32,35,36,38–44,48,50,52,55,58–60,65,78]. In order to further explore the results obtained, subgroup analyses were carried out on meta-analyses that contained at least 10 studies. The studies were grouped by type of surgery, the results of which can be found in S1 File. In addition, sources of herterogeneity were further explored using regression analyses, the results of which can be found in S1 File.

#### Depression and time to wound healing.

**Fig 4:**
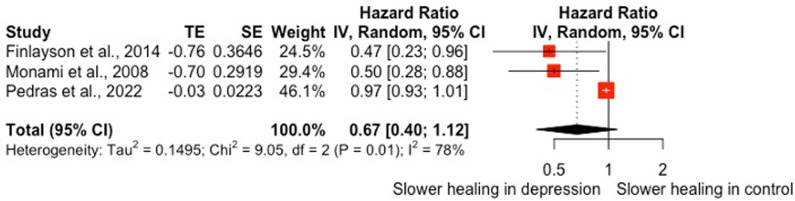
Forest plot of associations between depression and time to wound healing.

Three studies used Hazard Ratio’s (HRs) to explore whether depression influenced ulcer or [26,65] surgical wound healing [59]. All three studies identified depression using depressive symptom outcome measure scales. The studies were rated as strong [59,65] and moderate [26] in the quality appraisal. The pooled analysis did not indicate that depression was associated with increased time until wound healing HR = 0.67, 95% CI [0.40, 1.12], see Fig 4.

#### Depression and the likelihood of delayed wound healing.

**Fig 5:**
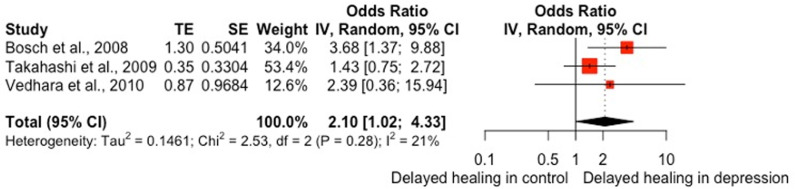
Forest plot of associations between depression and odds of delayed healing.

Three studies examined if individuals with high versus low depression had different odds of experimental [78] or ulcer [22,23] wounds healing. Two studies utilized validated measures of depressive symptoms (BDI, [78]; HADS, [22]) while one study utilized diagnosis of depression [23]. All studies were rated as moderate in the quality appraisal. The pooled analysis revealed that individuals with depression/high depressive symptoms were twice as likely to display slower wound healing OR = 2.10, 95% CI [1.02, 4.33], see Fig 5.

#### Depression and risk of wound complications/dehiscence.

**Fig 6:**
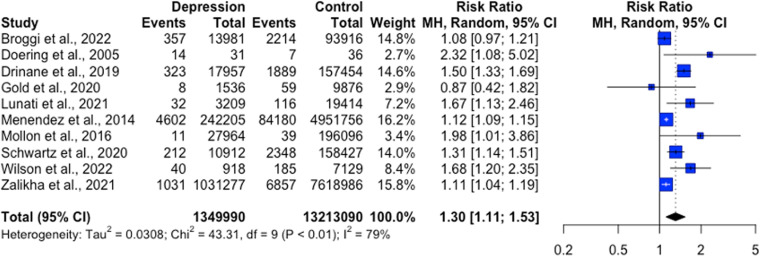
Forest plot of associations between depression and rates of wound complications/dehiscence.

Data from ten studies were combined to look at the relationship between depression and rates of wound complications [31,32,36,38,40,43,50,55] or wound dehiscence in surgical wounds [39,41]. Surgeries included ankle surgery [31,32], TKA and/or THA [36,38,39], TSA [40,41], spinal surgery [43]), CABG [50] and breast reconstruction after mastectomy [55]. Eight studies compared individuals with or without a depression diagnosis, except the studies by Mollon and colleagues [41] who compared participant with or without a history of clinical depression and the study by Doering and colleagues [50] which categorised participants based on their depressive scores on the Multiple Affect Adjective Check List [82]. The studies contributing to this analysis were rated moderate and strong in the quality appraisal. Overall, depression was associated with a greater risk of wound complications/dehiscence, RR = 1.30, 95% CI [1.11, 1.53], see Fig 6.

#### Depression and risk of wound infection.

**Fig 7:**
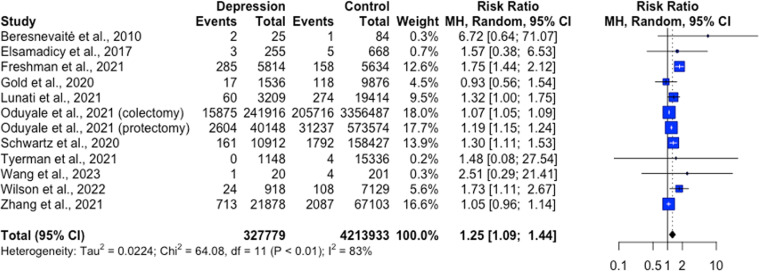
Forest plot of associations between depression and rates of wound infection.

We examined the association between depression and rates of wound infection in 11 studies [32,35,36,38,40,42,44,48,52,58,60]. Studies were surgical and included a range of surgeries, including cardiac, spinal surgery, arthroscopy surgeries and proctectomy. Nine of the included studies classified participants based on whethre they had a current or historic diagnosis of depression, while two used depressive symptom outcome measures to categorise participants [44,48]. The types of infection outcomes contributing in this statistical synthesis include general wound infection [58,60], SSI ([32,35,38,42,44], PJIs [36,40], deep sternal wound infection [52] and leg wound infections [48]. The quality of the studies included were rated as moderate and strong. This meta-analysis revealed that individuals with depression had a significantly higher risk of developing wound infections, compared to those without depression, RR = 1.25, 95% CI [1.09, 1.44], see Fig 7.

## Discussion

Anxiety and depression showed a mixed pattern of relationships with wound healing. Of the 21 studies that examined whether there was a relationship between anxiety and wound healing, four found an association, four showed mixed results while 13 demonstrated no significant relationship. The results of the meta-analyses did not indicate that anxiety was associated with slowed wound healing or increased the risk of wound complications (i.e., rate of complication, dehiscence, re-hospitalization).

Substantially more studies explored the relationship between wound healing and depression (k = 48). Nineteen studies found an association between depression and wound healing, 25 found no significant relationships and four found mixed results. Of the 19 studies that found an association between depression and wound healing outcomes, approximately half (k = 10) used diagnoses of depression and the other half (k = 9) used depressive symptom measures. One of the current meta-analytic outcomes indicated that depressed individuals did not have significantly longer time to heal, while, conversely, another meta-analytic outcome demonstrated that individuals with depression were more likely to experience delayed wound healing. Furthermore, depressed individuals overall had higher rates of wound complications and wound infections. Three studies indicated that depression was associated with slower changes in wound surface area (indicating slower healing). There was no evidence that depression was associated with clinician rating of healing status.

Findings from the narrative synthesis suggest more consistent results for depression than anxiety on wound healing outcomes. However, the results are mixed with approximately half of studies demonstrating an association between depression and poorer wound healing outcomes. The meta-analysis provide greater clarity. Whilst there were no significant effects for anxiety, significant effects were found for depression. Greater depression was associated with more wound complications, more infections and slower wound healing with significant but small effects observed.

The mechanism by which depression may impact wound healing remains to be elucidated. It is possible that depression may exert direct effects on physiological processes underlying wound healing. Depression has been shown to impair the immune response through various cellular, molecular and immunological processes [97]. Importantly, inflammation and HPA axis hyperactivity have been identified as key factors in the neurobiology of depression [98] as well as key elements in the wound healing process [4]. A recent animal study explored the mechanism by which depression influences wound healing in rats [99]. This study found that depression had effects on inflammation and delayed wound healing and that antidepressant treatment counteracted these effects. Notably, alleviating the inflammatory response was one of the mechanisms by which the chosen antidepressant worked [99]. However, as the authors note, the effect of inflammation on wound healing is complex. Some level of inflammation is helpful and necessary for wound healing, however the level inflammation associated with depression may have a deleterious effect.

Another potential mechanism through which depression may impact wound healing is through cognitive processes. The perseverative cognition hypothesis [100] posits that worry (a feature of anxiety) and rumination (a feature of depression) are types of perseverative cognition that are relevant factors in somatic health. The authors present evidence that such cognitions have physiological sequelae that are associated with adverse health outcomes. Within such a model, perseverative cognition could be a moderator of the effect of emotion on bodily systems. Perseverative cognition prolongs stress-related psychological and physiological activation, by reactivating responses after a stressor has been experienced, slowing recovery, or intensifying short-term responses.

A further plausible mechanism by which depression may influence wound healing is through depression symptoms influencing health-related behaviours. Symptoms of depression include low levels of energy, insight, motivation, initiation, psychomotor slowing and disturbance of sleep and appetite [101]. Such symptoms could impact wound healing. For example, it has been shown that relatively modest sleep disturbance and nutritional deficiencies can impede, and delay wound healing [102,103].; On the other hand, exercise has been shown to accelerate wound healing [10], but activity levels can be reduced in depression. Furthermore, depressed patients are three times more likely than nondepressed patients to be noncompliant with medical treatment [104], meaning that patients with depression may not be engaging in recommended wound care procedures.

A better understanding of the mechanisms behind an association between depression and wound healing could help in developing integrated psycho-medical interventions. For example, medications could be used to target inflammation coupled with psychological interventions that target specific depressive symptoms/behaviours (e.g., increase exercise) or target depression and reduce perseverative cognitions (e.g., metacognitive therapy; [105]). There is evidence that a range of different psychological interventions can improve the rate of wound healing [106]. With the strongest effects seen in relaxation interventions for surgical wounds [106].

The quality of the studies included in this review was variable, as judged by the risk of bias assessments. The primary studies included employed different methods, research designs, and made different analytic decisions. For example, three of the included ulcer studies used the HADS [84] in different ways: one study classified anxiety or depression status using a cut-off score of 11 [22], another used a cut-off score of 9 to indicate caseness [64], while a third analysed HADS as a continuous variable [67]. Such variation in analytic decisions complicates interpretation of depression effects across studies.

The way in which results were reported limited statistical synthesis, with less than half of the studies in the narrative review contributing to the meta-analysis. This was largely due to insufficient statistical detail being reported. This raises important concerns over generalizability and increases the chance of bias. However, all outcomes relevant to the research question were included in the narrative and tabular synthesis, in an attempt to provide an accurate and rounded overview of the literature.

The strengths of this review include a comprehensive literature search and the consideration of distinct wound outcomes and psychological factors. By considering anxiety and depression separately, this review provides clarity on the impact of different psychological factors, rather than considering generic emotional or psychological variables such as stress. Limitations of this review include a restriction to papers published in academic journals, papers published in English and it did not include searches of grey literature, introducing the possibility that relevant reports may have been missed. A limitation of this review is that it did not explore the effects of different anxiety disorders (e.g., generalised anxiety versus obsessive compulsive disorder). Rather it considered the concept of anxiety more broadly (i.e., presence or severity of anxiety symptoms, or presence or absence of an anxiety disorder).

Overall, the present study demonstrated an association between depression and poorer wound healing outcomes. Relationships between wound healing and depression are likely to be complex and reciprocal in real world settings. There was less evidence to indicate a relationship between anxiety and wound outcomes, although fewer studies have investigated this relationship. Clinicians should be aware that patients with depression may be at higher risk for poorer wound healing outcomes. Future studies should investigate the mechanisms behind the associations between depression and wound healing in order to facilitate appropriate interventions.

## Supporting information

S1 Table Prisma checklist. (DOCX)

S2 Table Search strategy. (DOCX)

S3 Table Quality ratings. (DOCX)

S1 File Subgroup and heterogeneity analyses. (DOCX)

## References

[1] Guest JF, Fuller GW, Vowden P. Cohort study evaluating the burden of wounds to the UK’s National Health Service in 2017/2018: update from 2012/2013. BMJ Open. 2020;10(12):e045253. doi: 10.1136/bmjopen-2020-045253 33371051 PMC7757484

[2] Järbrink K, Ni G, Sönnergren H, Schmidtchen A, Pang C, Bajpai R, et al. The humanistic and economic burden of chronic wounds: a protocol for a systematic review. Syst Rev. 2017;6(1):15. doi: 10.1186/s13643-016-0400-8 28118847 PMC5259833

[3] Guo S, Dipietro LA. Factors affecting wound healing. J Dent Res. 2010;89(3):219–29. doi: 10.1177/0022034509359125 20139336 PMC2903966

[4] Gouin J-P, Kiecolt-Glaser JK. The impact of psychological stress on wound healing: methods and mechanisms. Immunol Allergy Clin North Am. 2011;31(1):81–93. doi: 10.1016/j.iac.2010.09.010 21094925 PMC3052954

[5] Godoy LD, Rossignoli MT, Delfino-Pereira P, Garcia-Cairasco N, de Lima Umeoka EH. A comprehensive overview on stress neurobiology: Basic concepts and clinical implications. Front Behav Neurosci. 2018;12:127. doi: 10.3389/fnbeh.2018.00127 30034327 PMC6043787

[6] Müller MB, Landgraf R, Keck ME. Vasopressin, major depression, and hypothalamic-pituitary-adrenocortical desensitization. Biol Psychiatry. 2000;48(4):330–3. doi: 10.1016/s0006-3223(00)00886-6 10960167

[7] Gouin J-P, Carter CS, Pournajafi-Nazarloo H, Glaser R, Malarkey WB, Loving TJ, et al. Marital behavior, oxytocin, vasopressin, and wound healing. Psychoneuroendocrinology, 2010;35(7):1082–90. doi: 10.1016/j.psyneuen.2010.01.009 20144509 PMC2888874

[8] Arnold M, Barbul A. Nutrition and wound healing. Plast Reconstr Surg. 2006;117(7S):42S–58S. doi: 10.1097/01.prs.0000225432.17501.6c 16799374

[9] Benveniste K, Thut P. The effect of chronic alcoholism on wound healing. Proc Soc Exp Biol Med, 1981;166(4):568–75. 7194481 10.3181/00379727-166-41110

[10] Emery CF, Kiecolt-Glaser JK, Glaser R, Malarkey WB, Frid DJ. Exercise accelerates wound healing among healthy older adults: a preliminary investigation. J Gerontol A Biol Sci Med Sci. 2005;60(11):1432–6. doi: 10.1093/gerona/60.11.1432 16339330

[11] McDaniel JC, Browning KK. Smoking, chronic wound healing, and implications for evidence-based practice. J Wound Ostomy Cont Nursing. 2014;41(5):415–E412. doi: 10.1097/WON.0000000000000057 PMC4241583 25188797

[12] Mostaghimi L, Obermeyer W, Ballamudi B, Martinez-Gonzalez D, Benca R. Effects of sleep deprivation on wound healing. J Sleep Res. 2005;14(3):213–9. doi: 10.1111/j.1365-2869.2005.00455.x 16120095

[13] Walburn J, Vedhara K, Hankins M, Rixon L, Weinman J. Psychological stress and wound healing in humans: a systematic review and meta-analysis. J Psychosom Res. 2009;67(3):253–71. doi: 10.1016/j.jpsychores.2009.04.002 19686881

[14] Page MJ, McKenzie JE, Bossuyt PM, Boutron I, Hoffmann TC, Mulrow CD, et al. The PRISMA 2020 statement: an updated guideline for reporting systematic reviews. BMJ. 2021;372:n71. doi: 10.1136/bmj.n71 33782057 PMC8005924

[15] Bramer WM, Giustini D, de Jonge GB, Holland L, Bekhuis T. De-duplication of database search results for systematic reviews in EndNote. J Med Libr Assoc. 2016;104(3):240–3. doi: 10.3163/1536-5050.104.3.014 27366130 PMC4915647

[16] Thomas H, Ciliska D, Dobbins M. Quality assessment tool for quantitative studies. Toronto: Effective Public Health Practice Project, McMaster University; 2003.

[17] Armijo-Olivo S, Stiles CR, Hagen NA, Biondo PD, Cummings GG. Assessment of study quality for systematic reviews: a comparison of the Cochrane Collaboration Risk of Bias Tool and the Effective Public Health Practice Project Quality Assessment Tool: methodological research. J Eval Clin Pract, 2012;18(1):12–8. doi: 10.1111/j.1365-2753.2010.01516.x 20698919

[18] R Core Development Team. (2021). R: A Language and Environment for Statistical Computing. In (Version 4.1.2) R Foundation for Statistical Computing https://www.R-project.or

[19] Balduzzi S, Rücker G, Schwarzer G. How to perform a meta-analysis with R: a practical tutorial. Evid-Based Ment Health. 2019;22:153–160. doi: 10.1136/ebmental-2019-300117 31563865 PMC10231495

[20] Harrer M, Cuijpers P, Furukawa TA, Ebert DD (2021). Doing Meta-Analysis With R: A Hands-On Guide (1st ed.). Chapman & Hall/CRC Press. https://www.routledge.com/Doing-Meta-Analysis-with-R-A-Hands-On-Guide/Harrer-Cuijpers-Furukawa-Ebert/p/book/9780367610074

[21] The Cochrane Collaboration (2020) Review Manager (Revman) [Computer Program] Version 5.4.

[22] Vedhara K, Miles JN, Wetherell MA, Dawe K, Searle A, Tallon D, et al. Coping style and depression influence the healing of diabetic foot ulcers: observational and mechanistic evidence. Diabetologia. 2010;53(8):1590–8. doi: 10.1007/s00125-010-1743-7 20411235

[23] Takahashi PY, Kiemele LJ, Chandra A, Cha SS, Targonski PV. A retrospective cohort study of factors that affect healing in long-term care residents with chronic wounds. Ostomy Wound Manag. 2009;55(1):32–7. 19174587

[24] Tarp J, Dalene KE, Hansen BH, Steene-Johannessen J, Ekelund U. Double counting individuals in meta-analysis artificially inflates precision. Comment on “Device-measured light-intensity physical activity and mortality: A meta-analysis”. Scand J Med Sci Sports. 2020;30(6):1083–84. doi: 10.1111/sms.13693 32316073

[25] Jess P, von der Lieth L, Matzen P, Madsen P, Krag E, Knigge U, et al. The personality pattern of duodenal ulcer patients in relation to spontaneous ulcer healing and relapse. J Intern Med. 1989;226(6):395–400. doi: 10.1111/j.1365-2796.1989.tb01414.x 2489224

[26] Monami M, Longo R, Desideri CM, Masotti G, Marchionni N, Mannucci E. The diabetic person beyond a foot ulcer - Healing, recurrence, and depressive symptoms [Article]. J Am Podiatr Med Assoc. 2008;98(2):130–6. doi: 10.7547/0980130 18347122

[27] Gouin J-P, Kiecolt-Glaser JK, Malarkey WB, Glaser R. The influence of anger expression on wound healing. Brain Behav Immun. 2008;22(5):699–708. doi: 10.1016/j.bbi.2007.10.013 18078737 PMC2502071

[28] Koschwanez HE, Kerse N, Darragh M, Jarrett P, Booth RJ, Broadbent E (). Expressive writing and wound healing in older adults: a randomized controlled trial. Psychosom Med. 2013;75(6):581–90. doi: 10.1097/PSY.0b013e31829b7b2e 23804013

[29] Robles TF, Brooks KP, Pressman SD. Trait positive affect buffers the effects of acute stress on skin barrier recovery. Health Psychology. 2009;28(3):373–8. doi: 10.1037/a0014662 19450044 PMC2956507

[30] Buller LT, Best MJ, Klika AK, Barsoum WK. The influence of psychiatric comorbidity on perioperative outcomes following primary total hip and knee arthroplasty; a 17-year analysis of the national hospital discharge survey database. J Arthroplasty. 2015;30(2):165–170. doi: 10.1016/j.arth.2014.08.034 25267536

[31] Broggi MS, Tahmid S, Hurt J, Kadakia RJ, Bariteau JT, Coleman MM. Preoperative depression is associated with increased complications following ankle fracture surgery. Foot Ankle Spec. 2022. doi: 10.1177/19386400211065967 35037505

[32] Wilson JM, Schwartz AM, Farley KX, Bariteau JT. Preoperative depression influences outcomes following total ankle arthroplasty. Foot Ankle Spec. 2022;15(4):321–9. 10.1177/1938640020951657 32865018

[33] Bozic KJ, Lau E, Kurtz S, Ong K, Berry DJ, Bozic KJ, et al. Patient-related risk factors for postoperative mortality and periprosthetic joint infection in medicare patients undergoing TKA. Clin Orthop Relat Res. 2012;470(1):130–7. doi: 10.1007/s11999-011-2043-3 21874391 PMC3237966

[34] De Gouveia WM, Salem HS, Chen Z, Tarazi JM, Ehiorobo JO, Vakharia RM, et al. Increased in-hospital lengths of stay, readmission rates, complications, and costs in patients who have depressive disorders following primary total hip arthroplasty. Surg Technol Int. 2022;40:335–40. doi: 10.52198/22.STI.40.OS1548 35090180

[35] Freshman RD, Salesky M, Cogan CJ, Lansdown DA, Zhang AL. Association between comorbid depression and rates of postoperative complications, readmissions, and revision arthroscopic procedures after elective hip arthroscopy. Orthop J Sports Med. 2021;9(9):1–9. doi: 10.1177/23259671211036493 PMC8427924 34514010

[36] Gold PA, Garbarino LJ, Anis HK, Neufeld EV, Sodhi N, Danoff JR, et al. The cumulative effect of substance abuse disorders and depression on postoperative complications after primary total knee arthroplasty. J Arthroplasty. 2020;35(6S):S151–7. doi: 10.1016/j.arth.2020.01.027 32061474

[37] Pan X, Wang J, Lin Z, Dai W, Shi Z. Depression and anxiety are risk factors for postoperative pain-related symptoms and complications in patients undergoing primary total knee arthroplasty in the United States. J Arthroplasty. 2019;34(10):2337–46. doi: 10.1016/j.arth.2019.05.035 31229373

[38] Schwartz AM, Wilson JM, Farley KX, Roberson JR, Guild GN 3rd, Bradbury TL Jr. Modifiability of depression’s impact on early revision, narcotic usage, and outcomes after total hip arthroplasty: The impact of psychotherapy. The J Arthroplasty. 2020;35(10):2904–10. doi: 10.1016/j.arth.2020.05.021 32553794

[39] Zalikha AK, Karabon P, Hajj Hussein I, El-Othmani MM. Anxiety and depression impact on inhospital complications and outcomes after total knee and hip arthroplasty: A propensity score-weighted retrospective analysis. J Am Acad Orthop Surg, 2021;29(20):873–84. 10.5435/JAAOS-D-20-00721 34525481

[40] Lunati MP, Wilson JM, Farley KX, Gottschalk MB, Wagner ER. Preoperative depression is a risk factor for complication and increased health care utilization following total shoulder arthroplasty. J Shoulder Elbow Surg, 2021;30(1):89–96. 10.1016/j.jse.2020.04.015 33317706

[41] Mollon B, Mahure SA, Ding DY, Zuckerman JD, Kwon YW. The influence of a history of clinical depression on peri-operative outcomes in elective total shoulder arthroplasty: A ten-year national analysis. Bone Jt J. 2016;98-B(6), 818–24. 10.1302/0301-620X.98B6.37208 27235526

[42] Elsamadicy AA, Adogwa O, Lydon E, Sergesketter A, Kaakati R, Mehta AI, et al. Depression as an independent predictor of postoperative delirium in spine deformity patients undergoing elective spine surgery. J Neurosurg Spine. 2017;27(2):209–14. doi: 10.3171/2017.4.SPINE161012 28574333

[43] Menendez ME, Neuhaus V, Bot AGJ, Ring D, Cha TD. Psychiatric disorders and major spine surgery: Epidemiology and perioperative outcomes. Spine. 2014;39(2):E111–22. doi: 10.1097/BRS.0000000000000064 24108288

[44] Wang SK, Cui P, Wang DF, Wang P, Kong C, Lu SB. Preoperative Zung depression scale predicts outcomes in older patients undergoing short-segment fusion surgery for degenerative lumbar spinal disease. Eur Spine J. 2023;32(2):718–26. doi: 10.1007/s00586-022-07497-0 36562871

[45] George JM, Scott DS, Turner SP, Gregg JM (). The effects of psychological factors and physical trauma on recovery from oral surgery. J Behav Med. 1980;3(3):291–310. doi: 10.1007/BF00845053 7441729

[46] Kloostra PW, Eber RM, Wang H-L, Inglehart MR. Surgical versus non-surgical periodontal treatment: psychosocial factors and treatment outcomes. J Periodontol. 2006;77(7):1253–60. doi: 10.1902/jop.2006.050302 16805690

[47] Yahya BH, Chaushu G, Hamzani Y. Evaluation of wound healing following surgical extractions using the IPR scale. Int Dent J. 2021;71(2):133–9. doi: 10.1111/idj.12622 PMC9275323 33031642

[48] Beresnevaitė M, Benetis R, Taylor GJ, Jurėnienė K, Kinduris Š, Barauskienė V. Depression predicts perioperative outcomes following coronary artery bypass graft surgery. Scand Cardiovasc J. 2010;44(5):289–94. doi: 10.3109/14017431.2010.490593 21080846

[49] Doering LV, Martínez-Maza O, Vredevoe DL, Cowan MJ. Relation of depression, natural killer cell function, and infections after coronary artery bypass in women. Eur J Cardiovasc Nurs. 2008;7(1):52–8. doi: 10.1016/j.ejcnurse.2007.07.004 17716947 PMC2292641

[50] Doering LV, Moser DK, Lemankiewicz W, Luper C, Khan S. Depression, healing, and recovery from coronary artery bypass surgery. Am J Crit Care. 2005;14(4):316–24. doi: 10.4037/ajcc2005.14.4.316 15980423

[51] Scheier MF, Matthews KA, Owens JF, Schulz R, Bridges MW, Magovern GJ Sr, et al. Optimism and rehospitalization after coronary artery bypass graft surgery. Arch Int Med. 1999;159(8):829–35. doi: 10.1001/archinte.159.8.829 10219928

[52] Tyerman Z, Mehaffey JH, Hawkins RB, Dahl J, Narahari A, Chancellor WZ, et al. History of serious mental illness is a predictor of morbidity and mortality in cardiac surgery. Ann Thorac Surg. 2020;13. doi: 10.1016/j.athoracsur.2020.04.118 32544450

[53] Britteon P, Cullum N, Sutton M. Association between psychological health and wound complications after surgery. Br J Surg. 2017;104(6):769–76. doi: 10.1002/bjs.10474 28195304

[54] Broadbent E, Petrie KJ, Alley PG, Booth RJ. Psychological stress impairs early wound repair following surgery. Psychosom Med. 2003;65(5):865–9. doi: 10.1097/01.psy.0000088589.92699.30 14508033

[55] Drinane JJ, Pham TH, Schalet G, Rezak K. Depression is associated with worse outcomes among women undergoing breast reconstruction following mastectomy. J Plast Reconstr Aesthet Surg. 2019;72(8):1292–8. doi: 10.1016/j.bjps.2019.03.036 31056434

[56] Jovanovic K, Kalezic N, Sipetic Grujicic S, Zivaljevic V, Jovanovic M, Kukic B, et al. Preoperative anxiety is associated with postoperative complications in vascular surgery: A cross-sectional study. World J Surg. 2022;46(8):1987–96. doi: 10.1007/s00268-022-06575-0 35507076

[57] Kassahun WT, Mehdorn M, Wagner TC, Babel J, Danker H, Gockel I. The effect of preoperative patient-reported anxiety on morbidity and mortality outcomes in patients undergoing major general surgery. Sci Rep. 2022;12(1):6312. doi: 10.1038/s41598-022-10302-z 35428818 PMC9012824

[58] Oduyale OK, Eltahir AA, Stem M, Prince E, Zhang GQ, Safar B, Efron JE, et al. What does a diagnosis of depression mean for patients undergoing colorectal surgery? J Surg Res. 2021;260:454–61. 10.1016/j.jss.2020.11.006 33272593 PMC7959253

[59] Pedras S, Meira-Machado L, Couto de Carvalho A, Carvalho R, Pereira MG. Anxiety and/or depression: which symptoms contribute to adverse clinical outcomes after amputation? J Men Health. 2022;31(6):792–800. doi: 10.1080/09638237.2020.1836554 33100065

[60] Zhang GQ, Canner JK, Prince EJ, Stem M, Taylor JP, Efron JE, et al. History of depression is associated with worsened postoperative outcomes following colectomy. Colorectal Dis. 2021;23(10):2559–66. 10.1111/codi.15790 34166552

[61] Bui UT, Edwards H, Finlayson K. Identifying risk factors associated with infection in patients with chronic leg ulcers. Int Wound J, 2017;15(2):283–90. doi: 10.1111/iwj.12867 29250935 PMC7950101

[62] Bui UT, Finlayson K, Edwards H. Risk factors for infection in patients with chronic leg ulcers: A survival analysis. Int J Clin Pract. 2018;72(12):e13263. doi: 10.1111/ijcp.13263 30239088

[63] Chaby G, Senet P, Ganry O, Caudron A, Thuillier D, Debure C, et al. Prognostic factors associated with healing of venous leg ulcers: a multicentre, prospective, cohort study. Br J Dermatol. 2013;169(5):1106–13. doi: 10.1111/bjd.12570 23909381

[64] Cole-King A, Harding KG. Psychological factors and delayed healing in chronic wounds. Psychosom Med. 2001;63(2):216–20. doi: 10.1097/00006842-200103000-00004 11292268

[65] Finlayson KJ, Courtney MD, Gibb MA, O’Brien JA, Parker CN, Edwards HE. The effectiveness of a four-layer compression bandage system in comparison with Class 3 compression hosiery on healing and quality of life in patients with venous leg ulcers: a randomised controlled trial. Int Wound J. 2014;11(1):21–7. 10.1111/j.1742-481X.2012.01033.x 22716129 PMC7950767

[66] Melikian R, O’Donnell TF, Suarez L, Iafrati MD. Risk factors associated with the venous leg ulcer that fails to heal after 1 year of treatment. J Vasc Surg Venous Lymphat Disord. 2019;7(1):98–105. doi: 10.1016/j.jvsv.2018.07.014 30558732

[67] Walburn J, Weinman J, Norton S, Hankins M, Dawe K, Banjoko B, et al. Stress, illness perceptions, behaviors, and healing in venous leg ulcers: Findings from a prospective observational study. Psychosom Med. 2017;79(5):585–92. doi: 10.1097/PSY.0000000000000436 27941577 PMC5638426

[68] Levenstein S, Prantera C, Scribano ML, Varvo V, Berto E, Spinella S. Psychologic predictors of duodenal ulcer healing. J Clin Gastroenterol. 1996;22(2):84–89. doi: 10.1097/00004836-199603000-00002 8742643

[69] Onoyama A, Hoshiyama M, Yabe H. Relationship between psychological factors and wound occurrence in patients with peripheral arterial disease in the leg. Int J Low Extrem Wounds. 2020;1:1–8. doi: 10.1177/1534734620943813 32806973

[70] Udovichenko OV, Maximova NV, Amosova MV, Yunilaynen OA, Berseneva EA, Starostina EG. Prevalence and prognostic value of depression and anxiety in patients with diabetic foot ulcers and possibilities of their treatment. Curr Diabetes Rev. 2017;13(1):97–106. doi: 10.2174/1573399812666160523143354 27211285

[71] Tarrier N, Gregg L, Edwards J, Dunn K. The influence of pre-existing psychiatric illness on recovery in burn injury patients: the impact of psychosis and depression. Burns. 2005;31(1):45–49. doi: 10.1016/j.burns.2004.06.010 15639364

[72] Wilson ERH, Wisely JA, Wearden AJ, Dunn KW, Edwards J, Tarrier N. Do illness perceptions and mood predict healing time for burn wounds? A prospective, preliminary study. J Psychosom Res. 2011;71(5):364–6. doi: 10.1016/j.jpsychores.2011.05.009 21999981

[73] Glaser R, Kiecolt-Glaser JK, Marucha PT, MacCallum RC, Laskowski BF, Malarkey WB. Stress-related changes in proinflammatory cytokine production in wounds. Arch Gen Psychiatry. 1999;56(5):450–6. doi: 10.1001/archpsyc.56.5.450 10232300

[74] Yang EV, Bane CM, MacCallum RC, Kiecolt-Glaser JK, Malarkey WB, Glaser R. Stress-related modulation of matrix metalloproteinase expression. J Neuroimmunol. 2002;133(1-2):144–50. doi: 10.1016/s0165-5728(02)00270-9 12446017

[75] McGuire L, Heffner K, Glaser R, Needleman B, Malarkey W, Dickinson S, et al. Pain and wound healing in surgical patients. Ann Behav Med. 2006;31(2):165–72. doi: 10.1207/s15324796abm3102_8 16542131

[76] Wilson SJ, Andridge R, Peng J, Bailey BE, Malarkey WB, Kiecolt-Glaser JK. Thoughts after marital conflict and punch biopsy wounds: Age-graded pathways to healing. Psychoneuroendocrinology. 2017;85:6–13. doi: 10.1016/j.psyneuen.2017.07.489 28783508 PMC6555483

[77] Bosch JA, Engeland CG, Cacioppo JT, Marucha PT. Depressive symptoms predict mucosal wound healing. Psychosom Med. 2007;69(7):597–605. doi: 10.1097/PSY.0b013e318148c682 17766687

[78] Robles TF, Brooks KP, Kane HS, Schetter CD. Attachment, skin deep? Relationships between adult attachment and skin barrier recovery. Int J Psychophysiol. 2013;88(3):241–52. doi: 10.1016/j.ijpsycho.2012.04.007 22546664 PMC3467323

[79] Rietveld CA, Esko T, Davies G, Pers TH, Turley P, Benyamin B, et al. Common genetic variants associated with cognitive performance identified using the proxy-phenotype method. Proc Natl Acad Sci U S A. 2014;111(38):13790–4. doi: 10.1073/pnas.1404623111 25201988 PMC4183313

[80] Moerman N, van Dam FS, Muller MJ, Oosting H. The Amsterdam Preoperative Anxiety and Information Scale (APAIS). Anesth Analg. 1996;82(3):445–51. doi: 10.1097/00000539-199603000-00002 8623940

[81] Radloff LS. The CES-D scale: A self-report depression scale for research in the general population. Appl. Psychol Meas. 1977;1(3):385–401.

[82] Freedland KE, Skala JA, Carney RM, Raczynski JM, Taylor CB, Mendes de Leon CF, et al. The Depression Interview and Structured Hamilton (DISH): rationale, development, characteristics, and clinical validity. Psychosom Med. 2002;64(6):897–905. doi: 10.1097/01.psy.0000028826.64279.29 12461195

[83] Eysenck HJ. A short questionnaire for the measurement of two dimensions of personality. J Appl Psychol. 1958;42(1):14–7. doi: 10.1037/h0041738

[84] Zigmond AS, Snaith RP. The hospital anxiety and depression scale. Acta Psychiatr Scand. 1983;67:361–70. doi: 10.1111/j.1600-0447.1983.tb09716.x 6880820

[85] Zuckerman M, Lubin B, Robins S. Validation of the multiple affect adjective check list in clinical situations. J Consult Psychol. 1965;29(6):594. doi: 10.1037/h0022750 5846133

[86] Derogatis LR. SCL 90 R administration, scoring and procedures manual II for the revised version and other instruments of the psychopathology rating scale series Clin Psych Res. 1986.

[87] Zung WW. A self-rating depression scale. Arch Gen Psychiatry. 1965;12:63–70. doi: 10.1001/archpsyc.1965.01720310065008 14221692

[88] Spielberger CD, Gorsuch RL, Lushene R, Vagg PR, Jacobs GA. Manual for the State-Trait Anxiety Inventory. Consulting Psychologists Press. 1983.

[89] Beck AT, Steer RA, Brown GK. Manual for the Beck Depression Inventory-II. San Antonio, TX: Psychological Corporation. 1996.

[90] Sheikh JI, Yesavage JA. Geriatric Depression Scale (GDS): Recent evidence and development of a shorter version. Clin Gerontol. 1986.

[91] Dahlstrom WG, Welsh GS, Dahlstrom LE. An MMPI handbook. Vol. I. Clinical interpretation. University of Minnesota Press. 1972.

[92] Zung WW. A rating instrument for anxiety disorders. Psychosomatics. 1971;12(6):371–9. doi: 10.1016/s0033-3182(71)71479-0 5172928

[93] Beck AT, Steer RA. Beck Anxiety Inventory Manual. Psychological Corporation. 1993.

[94] Beck AT, Steer RA, Carbin MG. Psychometric properties of the Beck Depression Inventory: Twenty-five years of evaluation. Clin Psychol Rev. 1988;8(1):77–100. doi: 10.1016/0272-7358(88)90050-5

[95] Fraley RC, Waller NG, Brennan KA. An item response theory analysis of self-report measures of adult attachment. J Pers Soc Psychol. 2000;78(2):350–65. doi: 10.1037//0022-3514.78.2.350 10707340

[96] Watson D, Clark LA, Tellegen A. Development and validation of brief measures of positive and negative affect: the PANAS scales. J Pers Soc Psychol. 1988;54(6):1063. 3397865 10.1037//0022-3514.54.6.1063

[97] Reiche EMV, Nunes SOV, Morimoto HK. Stress, depression, the immune system, and cancer. Lancet Oncol. 2004;5(10):617–625. doi: 10.1016/S1470-2045(04)01597-9 15465465

[98] Dean J, Keshavan M. The neurobiology of depression: An integrated view. Asian J Psychiatr. 2017;27:101–11. doi: 10.1016/j.ajp.2017.01.025 28558878

[99] Jian J, Yi-Heng H, Bang-Hui Z, Jian-Hua C, Xu-Dong Z, Shi-Chu X, et al. Effects of depression on healing and inflammatory responses of acute wounds in rats. Wound Repair Regen. 2019;27(5):462–9. doi: 10.1111/wrr.12726 31077486

[100] Brosschot JF, Gerin W, Thayer JF. The perseverative cognition hypothesis: a review of worry, prolonged stress-related physiological activation, and health. J Psychosom Res. 2006;60(2):113–24. doi: 10.1016/j.jpsychores.2005.06.074 16439263

[101] Ishizaki J, Mimura M. Dysthymia and apathy: diagnosis and treatment. Depress Res Treat. 2011;2011:893905. doi: 10.1155/2011/893905 21747995 PMC3130974

[102] Smith TJ, Wilson M, Karl JP, Orr J, Smith C, Cooper A, et al. Impact of sleep restriction on local immune response and skin barrier restoration with and without “multinutrient” nutrition intervention. J Appl Physiol. 2018;124(1):190–200. doi: 10.1152/japplphysiol.00547.2017 28912361

[103] Stechmiller JK. Understanding the role of nutrition and wound healing. Nutr Clin Pract. 2010;25(1):61–8. doi: 10.1177/0884533609358997 20130158

[104] DiMatteo MR, Lepper HS, Croghan TW. Depression is a risk factor for noncompliance with medical treatment: meta-analysis of the effects of anxiety and depression on patient adherence. Arch Intern Med, 2000;160(14):2101–7. doi: 10.1001/archinte.160.14.2101 10904452

[105] Wells A. Metacognitive therapy for anxiety and depression. New York: Guilford Press. 2009.

[106] Robinson H, Norton S, Jarrett P, Broadbent E. The effects of psychological interventions on wound healing: A systematic review of randomized trials. Br J Health Psychol. 2017;22(4):805–35. doi: 10.1111/bjhp.12257 Epub 2017 Jul 3. . 28670818

