# Supplementary material for: Relationships between anxiety, depression and wound healing outcomes in adults: A systematic review and meta-analysis
Source: PLoS One. 2025 May 20;20(5):e0309683. doi: 10.1371/journal.pone.0309683 (PMC12091741; doi:10.1371/journal.pone.0309683)
Supplement: S2 Table — (DOCX) [file pone.0309683.s002.docx]

Appendix 1: Full Search Strategy from Electronic Databases

Table S1

*Details of searches run on 06^th^ March 2023*

| **#** | **Query** |
| --- | --- |
|  | **CINAHL Plus** |
| S1 | phq-9 |
| S2 | ces-d |
| S3 | "beck depression inventory" |
| S4 | "negative affect" |
| S5 | "low mood" |
| S6 | "depressive disorder" |
| S7 | "depressive symptoms" |
| S8 | depression |
| S9 | (MH "Depression") |
| S10 | (MH "Self-Rating Depression Scale") |
| S11 | "gad-7" |
| S12 | (MH "Worry") |
| S13 | "anxiety symptoms" |
| S14 | (MH "Anxiety") |
| S15 | Neuroticism |
| S16 | hospital anxiety and depression scale" |
| S17 | "beck anxiety inventory" |
| S18 | "trait anxiety inventory" |
| S19 | "trait anxiety" |
| S20 | worry |
| S21 | "anxiety disorder" |
| S22 | anxiety |
| S23 | S1 OR S2 OR S3 OR S4 OR S5 OR S6 OR S7 OR S8 OR S9 OR S10 OR S11 OR S12 OR S13 OR S14 OR S15 OR S16 OR S17 OR S18 OR S19 OR S20 OR S21 OR S22 |
| S24 | (MM "Wound Care") |
| S25 | (MM "Wound Healing") |
| S26 | (MM "Wounds and Injuries") |
| S27 | "tape stripping" |
| S28 | "suction blister" |
| S29 | "fracture healing" |
| S30 | (MH "Fracture Healing") |
| S31 | "burn injury" |
| S32 | (MH "Burns") |
| S33 | "postoperative complications" |
| S34 | (MH "Postoperative Complications") |
| S35 | "surgical recovery" |
| S36 | "surgical site infection" |
| S37 | (MH "Surgical Wound Infection") |
| S38 | (MH "Surgical Wound") |
| S39 | ulcer |
| S40 | (MH "Ulcer") |
| S41 | "punch biopsy" |
| S42 | transepidermal water loss |
| S43 | "Re-Epithelialization" |
| S44 | "wound healing" |
| S45 | S24 OR S25 OR S26 OR S27 OR S28 OR S29 OR S30 OR S31 OR S32 OR S33 OR S34 OR S35 OR S36 OR S37 OR S38 OR S39 OR S40 OR S41 OR S42 OR S43 OR S44 |
| S46 | S23 AND S45 |
| S47 | S23 AND S45 |
| Added limits: | Academic Journals, all adult, English |
|  | **Embase** |
| 1 | anxiety/ |
| 2 | anxiety.mp. |
| 3 | anxiety assessment/ |
| 4 | anxiety disorder/ |
| 5 | Beck Anxiety Inventory/ |
| 6 | Depression, Anxiety and Stress Scale/ |
| 7 | Generalized Anxiety Disorder-7/ |
| 8 | Hamilton Anxiety Scale/ |
| 9 | Hospital Anxiety and Depression Scale/ |
| 10 | Self-rating Anxiety Scale/ |
| 11 | State Trait Anxiety Inventory/ |
| 12 | Zung Self Rating Anxiety Scale/ |
| 13 | worry.mp. |
| 14 | neuroticism.mp. |
| 15 | depression/ |
| 16 | depression.mp. |
| 17 | Center for Epidemiological Studies Depression Scale/ |
| 18 | depression assessment/ |
| 19 | depression inventory/ |
| 20 | Geriatric Depression Scale/ |
| 21 | Montgomery Asberg Depression Rating Scale/ |
| 22 | Zung Self Rating Depression Scale/ |
| 23 | depressive symptoms.mp. |
| 24 | low mood.mp. |
| 25 | negative affectivity.mp. |
| 26 | wound healing/ |
| 27 | wound healing.mp. |
| 28 | surgical recovery.mp. |
| 29 | fracture healing/ |
| 30 | Re-epithelialization.mp. |
| 31 | skin water loss/ |
| 32 | tape stripping.mp. |
| 33 | punch biopsy/ |
| 34 | ulcer healing/ |
| 35 | ulcer healing rate/ |
| 36 | burn/ |
| 37 | suction blister.mp. |
| 38 | postoperative complication/ |
| 39 | 1 or 2 or 3 or 4 or 5 or 6 or 7 or 8 or 9 or 10 or 11 or 12 or 13 or 14 or 15 or 16 or 17 or 18 or 19 or 20 or 21 or 22 or 23 or 24 or 25 |
| 40 | 26 or 27 or 28 or 29 or 30 or 31 or 32 or 33 or 34 or 35 or 36 or 37 or 38 |
| 41 | 39 and 40 |
| 42 | limit 41 to (animals or animal studies) |
| 43 | limit 42 to conference abstracts |
| 44 | limit 41 to ("reviews (maximizes sensitivity)" or "reviews (maximizes specificity)" or "reviews (best balance of sensitivity and specificity)") |
| 45 | limit 41 to conference abstracts |
| 46 | limit 41 to (embryo <first trimester> or infant <to one year> or child <unspecified age> or preschool child <1 to 6 years> or school child <7 to 12 years> or adolescent <13 to 17 years>) |
| 47 | limit 41 to (afrikaans or albanian or arabic or armenian or azerbaidzhani or basque or belorussian or bengali or bulgarian or burmese or bosnian or catalan or chinese or croatian or czech or danish or dutch or esperanto or estonian or finnish or french or gallegan or georgian or german or greek or hebrew or hindi or hungarian or icelandic or indonesian or irish gaelic or italian or japanese or korean or latvian or lithuanian or macedonian or malay or maori or mongolian or norwegian or persian or polish or polyglot or portuguese or pushto or romanian or russian or scottish gaelic or serbian or sinhalese or slovak or slovene or spanish or swedish or tagalog or thai or turkish or ukrainian or urdu or uzbek or vietnamese) |
| 48 | limit 41 to (meta analysis or "systematic review") |
| 49 | limit 41 to ("qualitative (maximizes sensitivity)" or "qualitative (maximizes specificity)" or "qualitative (best balance of sensitivity and specificity)") |
| 50 | 41 not (42 or 43 or 44 or 45 or 46 or 47 or 48 or 49) |
|  | **Medline** |
| 1 | Anxiety/ |
| 2 | Anxiety Disorders/ |
| 3 | worry.mp. |
| 4 | Trait anxiety.mp. |
| 5 | Neuroticism/ |
| 6 | beck anxiety inventory.mp. |
| 7 | (Hospital Anxiety and Depression Scale).mp. |
| 8 | State-Trait Anxiety Inventory.mp. |
| 9 | GAD-7.mp. |
| 10 | hamilton anxiety scale.mp. |
| 11 | Penn State Worry Questionnaire.mp. |
| 12 | Depression/ |
| 13 | Affective Symptoms/ |
| 14 | Mood Disorders/ |
| 15 | Depressive Disorder/ |
| 16 | low mood.mp. |
| 17 | negative affectivity.mp. |
| 18 | beck depression inventory.mp. |
| 19 | Patient Health Questionnaire/ |
| 20 | CES-D.mp. |
| 21 | Wound Healing/ |
| 22 | wound healing.mp. |
| 23 | Re-Epithelialization/ |
| 24 | Transepidermal water loss.mp. |
| 25 | punch biopsy.mp. |
| 26 | Ulcer/ |
| 27 | Surgical Wound Infection/ |
| 28 | Postoperative Complications/ |
| 29 | Burns/ |
| 30 | surgical recovery.mp. |
| 31 | suction blister.mp. |
| 32 | tape stripping.mp. |
| 33 | Fracture Healing/ |
| 34 | 1 or 2 or 3 or 4 or 5 or 6 or 7 or 8 or 9 or 10 or 11 or 12 or 13 or 14 or 15 or 16 or 17 or 18 or 19 or 20 |
| 35 | 21 or 22 or 23 or 24 or 25 or 26 or 27 or 28 or 29 or 30 or 31 or 32 or 33 |
| 36 | 34 and 35 |
| 37 | limit 36 to ("all infant (birth to 23 months)" or "all child (0 to 18 years)" or "newborn infant (birth to 1 month)" or "infant (1 to 23 months)" or "preschool child (2 to 5 years)" or "child (6 to 12 years)" or "adolescent (13 to 18 years)") |
| 38 | limit 36 to (animals or "article reviews (acp journal club)" or "review articles" or "article reviews (dare)") |
| 39 | limit 36 to (afrikaans or albanian or arabic or armenian or azerbaijani or belorussian or bengali or bosnian or bulgarian or burmese or catalan or chinese or croatian or czech or danish or dutch or esperanto or estonian or finnish or flemish or french or gaelic, scots or georgian or german or greek or hausa or hebrew or hindi or hungarian or icelandic or indonesian or interlingua or italian or japanese or kirghiz or korean or latin or latvian or lithuanian or macedonian or malay or marathi or masai or multilingual or norwegian or persian or polish or portuguese or pushto or rumanian or russian or serbian or slovak or slovene or spanish or swahili or swedish or tagalog or tamil or telugu or thai or turkish or ukrainian or undetermined or urdu or vietnamese or welsh) |
| 40 | limit 36 to (address or autobiography or bibliography or comment or congress or dataset or dictionary or directory or interactive tutorial or lecture or legal case or letter or meta analysis or news or patient education handout or periodical index or personal narrative or portrait or "review" or "systematic review") |
| 41 | 36 not (37 or 38 or 39 or 40) |
|  | **PsycINFO** |
| 1 | Anxiety/ |
| 2 | Anxiety Disorders/ |
| 3 | worry.mp. |
| 4 | Trait anxiety.mp. |
| 5 | Neuroticism/ |
| 6 | beck anxiety inventory.mp. |
| 7 | State-Trait Anxiety Inventory.mp. |
| 8 | GAD-7.mp. |
| 9 | hamilton anxiety scale.mp. |
| 10 | Penn State Worry Questionnaire.mp. |
| 11 | exp "Depression (Emotion)"/ |
| 12 | depression.mp. |
| 13 | exp Zungs Self Rating Depression Scale/ |
| 14 | exp Beck Depression Inventory/ |
| 15 | low mood.mp. |
| 16 | negative emotions/ |
| 17 | negativism/ |
| 18 | exp Wounds/ |
| 19 | wound healing.mp. |
| 20 | postsurgical complications/ |
| 21 | ulcer.mp. |
| 22 | fracture.mp. |
| 23 | exp Skin Resistance/ |
| 24 | tape stripping.mp. |
| 25 | suction blister.mp. |
| 26 | Transepidermal water loss.mp. |
| 27 | skin barrier recovery.mp. |
| 28 | punch biopsy.mp. |
| 29 | exp Burns/ |
| 30 | 1 or 2 or 3 or 4 or 5 or 6 or 7 or 8 or 9 or 10 or 11 or 12 or 13 or 14 or 15 or 16 or 17 |
| 31 | 19 or 20 or 21 or 22 or 23 or 24 or 25 or 26 or 27 or 28 or 29 |
| 32 | 30 and 31 |
| 33 | limit 32 to animal |
| 34 | limit 32 to ("0800 literature review" or "0830 systematic review" or 1000 mathematical model or 1200 meta analysis or 1300 metasynthesis or 1600 qualitative study) |
| 35 | limit 32 to (100 childhood <birth to age 12 yrs> or 120 neonatal <birth to age 1 mo> or 140 infancy <2 to 23 mo> or 160 preschool age <age 2 to 5 yrs> or 180 school age <age 6 to 12 yrs> or 200 adolescence <age 13 to 17 yrs>) |
| 36 | limit 32 to (abstract collection or bibliography or "column/opinion" or dissertation or encyclopedia entry or interview or obituary or poetry or publication information or reprint or retraction or review-book or review-media or review-software & other or reviews) |
| 37 | 32 not (33 or 34 or 35 or 36) |
|  | **Web of Science** |
| #1 | ((((TS=(anxiety)) OR TS=(worry )) OR TS=(Neuroticism) OR TS = (Trait NEAR anxiety) OR TS = (Anxiety NEAR disorder))) |
| #2 | (TS=(depression) OR TS=(low NEAR mood) OR TS=(negative NEAR affect) OR TS=(mood NEAR disorder) OR TS=(depressive NEAR disorder)) |
| #3 | (TS=(wound NEAR healing) OR TS=(surgical NEAR recovery) OR TS=(skin NEAR healing) OR TS=(experimental NEAR wound) AND TS=(wound NEAR infection) OR TS=(Re-Epithelialization) OR TS=(punch NEAR biopsy) OR TS=(fracture NEAR healing) ) |
|  | ((#1) OR #2) AND #3 |
|  | ((#1) OR #2) AND #3 and Review Articles or Proceedings Papers or Book Chapters (Exclude – Document Types) |
|  | ((#1) OR #2) AND #3 and Review Articles or Proceedings Papers or Book Chapters (Exclude – Document Types) and English (Languages) |
