# Supplementary material for: Relationships between anxiety, depression and wound healing outcomes in adults: A systematic review and meta-analysis
Source: PLoS One. 2025 May 20;20(5):e0309683. doi: 10.1371/journal.pone.0309683 (PMC12091741; doi:10.1371/journal.pone.0309683)
Supplement: S1 File — (DOCX) [file pone.0309683.s004.docx]

**Supplementary File 1 – Further Exploratory Analyses: Subgroup and Regression Analyses**

**Subgroup analyses**

In order to further explore the results obtained, subgroup analyses were carried out on meta-analyses that contained at least 10 studies. The studies were grouped by type of surgery.

**Depression and risk of wound complications/dehiscence – Subgroup Analysis**

Data from ten studies was combined to examine the impact of depression on rate of wound complications or dehiscence in surgical wounds. Seven of the studies were grouped as surgeries on joints, namely ankle surgery (Broggi et al., 2022; Wilson et al., 2022), TKA and/or THA (Gold et al., 2020; Schwartz et al., 2020; Zalikha et al., 2021), TSA (Lunati et al., 2021; Mollon et al., 2016). The other studies were considered separately since they looked at wounds from different types of surgeries, namely spinal surgery (Menendez et al., 2014), CABG (Doering et al., 2005) and breast reconstruction after mastectomy (Drinane et al., 2019). The studies were grouped as joint replacement surgery (k = 7) or other type of surgery (k = 3).

Depression was associated with a greater risk of wound complications/dehiscence in the joint surgery subgroup , RR = 1.28, 95% CI [1.04, 1.58], but not in the other types of surgeries, RR = 1.39, 95% CI [0.67, 2.91]. There was no statistically significant difference between the subgroups, X^2^ (1) = 0.20, p =.066.

S1 Figure 1 *Forest plot of associations between depression and rates of wound*
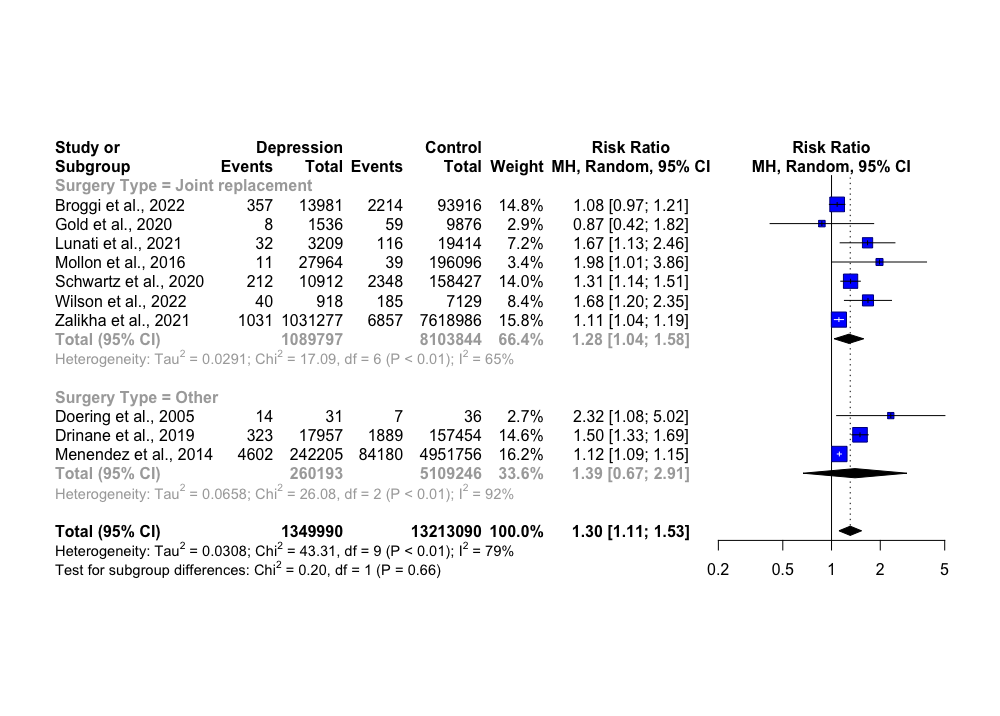
*complications/dehiscence - subgroup analysis by surgery type*

**Depression and risk of wound Infection – Subgroup Analysis**

Data from 11 studies were combined to examine the impact of depression on rate of wound infection in surgical wounds. Studies were grouped into cardiac (Beresnevaitė et al., 2010; Tyerman et al., 2021); spinal (Elsamadicy et al., 2017; Wang et al., 2023); joint replacement (Freshman et al., 2021; Gold et al., 2020; Lunati et al., 2021; Schwartz et al., 2020;; Wilson et al., 2022) and colectomy/protectomy surgeries (Oduyale et al., 2021; Zhang et al., 2021).

Depression was associated with a greater risk of infection in the joint surgery subgroup , RR = 1.42, 95% CI [1.08, 1.86]. However, was not significantly associated with risk of infections in the other subgroups namely cardiac surgery RR = 3.70, 95% CI [0.00, 27.54]; spinal surgery; RR = 1.81, 95% CI [0.12, 28.32]; or colectomy/proctectomy, RR = 1.11, 95% CI [0.93, 1.31].

There was a statistically significant difference between the subgroups, X^2^ (3) = 12.38, p <0.01.

S1 Figure 2 *Forest plot of associations between depression and rates of wound infection - subgroup analysis by surgery type*


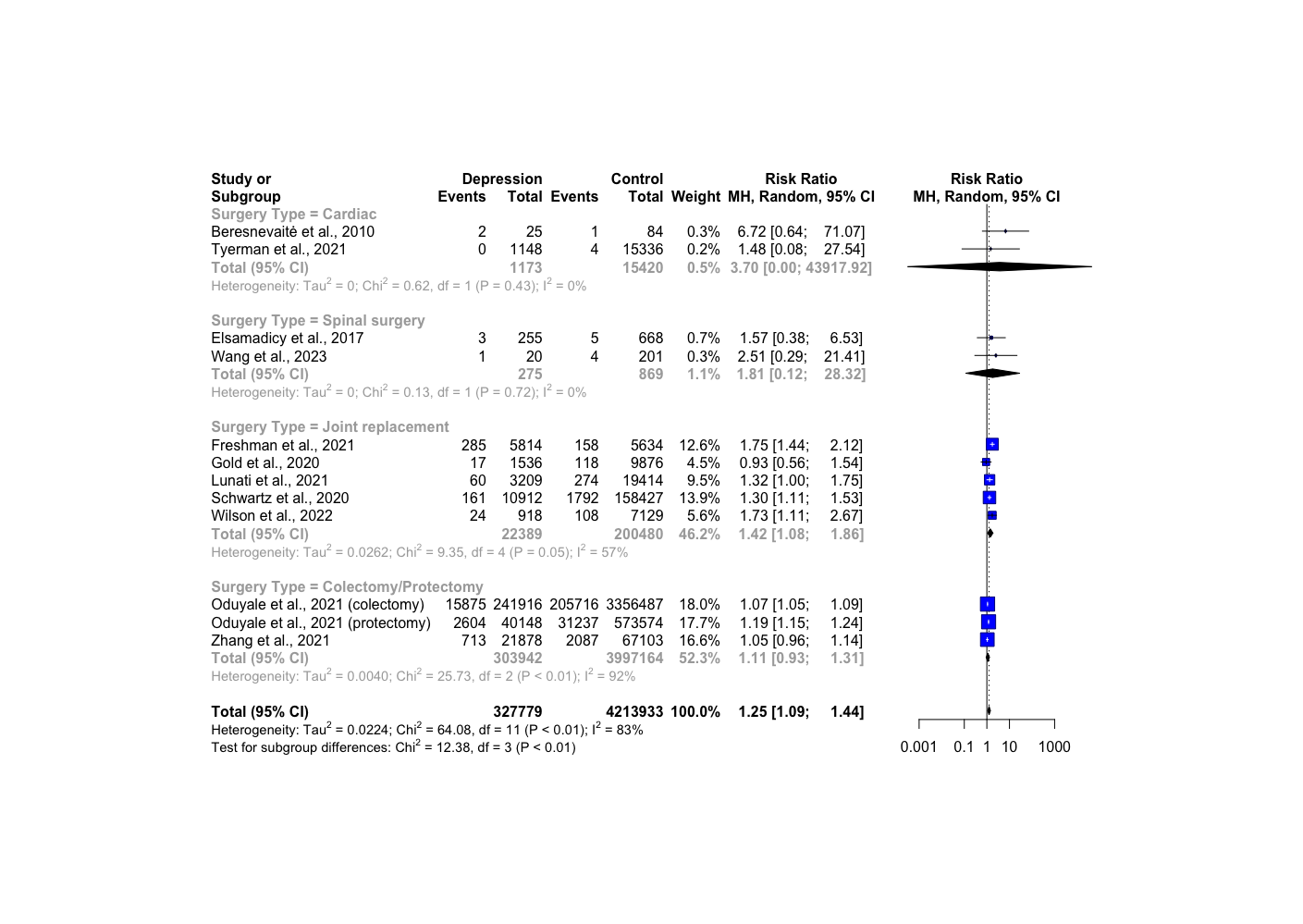


**Exploring Heterogeneity post hoc using regression**

Substantial heterogeneity was noted within meta-analytic results. Therefore, in the meta-analytic syntheses that exceeded 10 studies, heterogeneity was explored using regression analysis. Regression analyses were conducted separately for two predictor variables: 1) the quality rating of the study (i.e., strong, moderate or weak), and 2) the method of classifying depression (i.e., the presence or absence of a diagnosis of depression or a depressive symptoms outcome measure).

In the meta analysis that explored the relationship between depression and risk of wound complications/dehiscence, substantial heterogeneity was identified I^2^ = 79.2%, 95% CI [62.4%, 88.5%]. The quality of the studies (rated as weak, moderate, or strong) did not significantly explain the heterogeneity in the analysis, R^2^ = 0, *F*(1, 8) = 0.04, *p* = .85., nor did the measure of depression (symptom measure or diagnosis)  R^2^ = 9.64, *F*(1, 8) = 1.85, *p* = .21.

In the meta analysis that examined the association between depression and wound infection, substantial heterogeneity was identified  I^2^ = 82.8%,  95% CI [71.3%, 89.7%]. The quality of the study (weak, moderate, or strong) did not significantly explain study heterogeneity R^2^ = 1.61, *F*(1, 10) = 1.51, *p* = .25, nor did the measure of depression (symptom measure or diagnosis), R^2^ = 2.18, *F*(1, 10) = 2.48, *p* = .15.
